# Remote C─H Bond Activation via Enantioselective Carbopalladation and 1,4‐Pd Migration Cascade Process

**DOI:** 10.1002/advs.202406443

**Published:** 2024-09-03

**Authors:** Bing Xu, Danting Ji, Zhan‐Ming Zhang, Junliang Zhang

**Affiliations:** ^1^ Department of Chemistry Fudan University 2005 Songhu Road Shanghai 200438 China; ^2^ Zhuhai Fudan Innovation Institute Hengqing District Zhuhai 519000 China; ^3^ Shanghai Key Laboratory of Green Chemistry and Chemical Processes School of Chemistry and Molecular Engineering East China Normal University 3663 N. Zhongshan Road Shanghai 200062 China; ^4^ Fudan Zhangjiang Institute Shanghai 201203 China; ^5^ School of Chemistry and Chemical Engineering Henan Normal University Xinxiang Henan 453007 China

**Keywords:** 1,4‐Pd migration, asymmetric catalysis, cascade reaction, C─H bond activation, palladium

## Abstract

Carbopalladation‐initiated cascade reaction involving 1,4‐Pd migration is a straightforward and powerful approach to activate remote C─H bond, forging versatile fused polycyclic compounds containing fluorene fragment which are highly valuable synthetic targets. However, its asymmetric variants pose considerable challenges and have not been explored. Here the first asymmetric palladium‐catalyzed tandem carbopalladation is reported, 1,4‐Pd migration reaction of ortho‐iodophenol‐derived allyl ether under mild conditions, allowing the transformation of a wide range of substrates in good to excellent enantioselectivities, and providing a facile and straight forward access to tetracyclic dihydroindeno[1,2,3‐de]chromene bearing a chiral fluorene skeleton. A good functional group tolerance, high stereoselectivity, as well as the good chiroptical properties (high fluorescence quantum yields, circular dichroism) of the products make this approach highly attractive. Moreover, density functional theory (DFT) calculations indicate that the protonation of five‐membered palladacycle intermediate is more favorable rather than its direct reductive elimination process.

## Graphical abstract

A highly enantioselective palladium‐catalyzed tandem carbopalladation, 1,4‐Pd migration reaction of ortho‐iodophenol‐derived allyl ether is established, offering an unprecedented efficient route for the selective synthesis of tetracyclic dihydroindeno[1,2,3‐de]chromene bearing a chiral fluorene skeleton. This process features broad functional group compatibility, mild reaction conditions, excellent chemo and enantioselectivities, as well as good chiroptical properties.

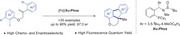

## 1 Introduction

**Figure 1:**
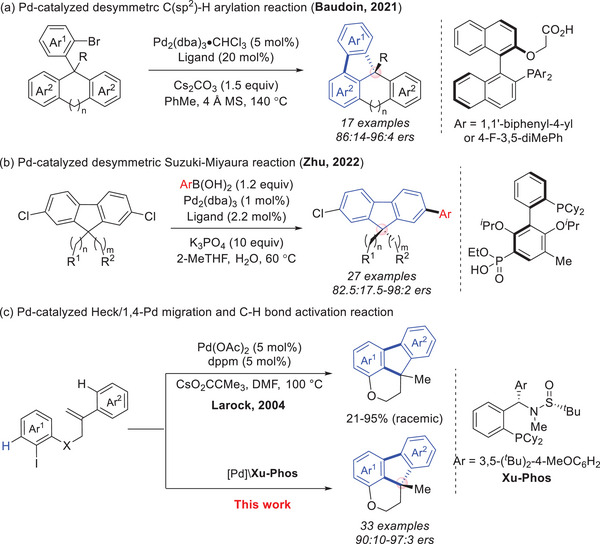
Various Pd‐catalyzed reactions to construct chiral polycyclic fragments.

Fluorene fragment, an important class of polycyclic aromatic hydrocarbons, featuring unique optical properties and biological activities on account of its special rigid biphenyl structure and large conjugated system,^[^ ^1^, ^2^ ^]^ is ubiquitous applicable in functional materials and medicinal chemistry.^[^ ^3^, ^4^, ^5^, ^6^, ^7^, ^8^ ^]^ Given the important influence of fluorene skeleton, the exploration of its asymmetric variants, to which has been devoted much efforts, continue to meet with limited success. The vast majority of these enantioenriched fluorene molecules were obtained on a small scale through chromatographic separation, which has significantly hampered research efforts on the application of these chiral compounds. Recently, an elegant Pd‐catalyzed desymmetric C(sp^2^)‐H arylation protocol has been developed by the group of Baudoin, delivering diverse polycyclic compounds bearing the chiral fluorene ring (**Figure** **1**a).^[^ ^9^ ^]^ Afterward, Zhu and co‐workers accomplished a desymmetric Suzuki−Miyaura reaction that established chirality on fluorene skeleton (Figure 1b).^[^ ^10^ ^]^ However, these exsiting methods require a pre‐installed carbon quaternary center on the substrates,^[^ ^11^ ^]^ and strategies for the direct construction a carbon quaternary center of chiral fluorenes from simple, readily available materials remain a considerable challenge but are highly desirable.

In 2004, Larock reported an elegant palladium‐catalyzed tandem carbopalladation, 1,4‐Pd migration and C─H bond activation process for the direct preparation of fluorene containing fused polycycles, which is an important carbon‐based molecular architecture found in graphene substructures and ribbons, leading to the formation of two kinds of carbon‐carbon bonds (C(*sp^2^*)‐C(*sp^3^*), C(*sp^2^*)‐C(*sp^2^*)) with simultaneous creation of a quaternary carbon center (Figure 1c, top).^[^ ^12^ ^]^ Since Larock's seminal contribution, the formation of plenty of valuable fluorene containing polycyclic heterocycles has been reported by the groups of Zhu,^[^ ^13^ ^]^ Loh and Xu,^[^ ^14^ ^]^ Ji and Wang,^[^ ^15^ ^]^ using the remote C─H bond functionalization via aforementioned similar cascade reaction. Although an array of privileged fused polycycles were prepared by those cascade reactions,^[^ ^12^, ^16^ ^]^ to the best of our knowledge, no successful exploration of its asymmetric variants is currently known. However, the selectivity of many different C─H bonds of aromatic ring which may lead to various competitive side reactions, i.e, domino Heck/intramolecular C─H alkylation reactions to form spirobicycles,^[^ ^17^, ^18^, ^19^, ^20^, ^21^, ^22^ ^]^ reductive Heck reaction^[^ ^23^, ^24^, ^25^ ^]^ and carboiodination reaction,^[^ ^26^, ^27^, ^28^, ^29^ ^]^ making the development of an enantioselective version of Larock's seminal work extremely challenging. Very recently, we realized the palladium catalyzed enantioselective cascade Heck/C─H bond activation reaction, leading to various spirobicycles, in which we observed the alkene insertion step is reversible at high temperatures.^[^ ^17^ ^]^ Herein, a Pd/**Xu‐Phos** catalyzed remote C─H bond activation via enantioselective carbopalladation and 1,4‐Pd migration cascade process was established, allowing expedient access to a wide array of dihydroindeno[1,2,3‐*de*]chromene derivatives bearing a chiral fluorene skeleton from simple linear precursors (Figure 1c, bottom).

## 2 Results and Discussion

We first investigated the asymmetric carbopalladation‐initiated involving 1, 4‐palladium migration cascade reaction of the easily accessible 1‐iodo‐2‐((3‐phenylbut‐3‐en‐1‐yl)oxy)benzene (**1a**) under the conditions by using Pd_2_(dba)_3_ and ***N*‐Me‐Xu3** catalytic system we developed previously in the related studies^[^ ^28^, ^29^ ^]^ with various bases in MTBE at 100 °C, but the desired **2a** was not formed and almost all the raw material **1a** was recoverd (more detail see the supplemental information; Table S1, Supporting Information). To our delight, using CsOAc as base, the target product **2a** was obtained in 41% yield with 89.5:10.5 er (**Table** **1**, entry 1), which demonstrated a remarkable effect of the base on the reactivity. Encouraged by this finding, we then conducted the reaction with different solvents. The use of Et_2_O and *^i^* Pr_2_O as solvents resulted in either slightly decreased yield or enantioselectivity (Table 1, entries 2–3). When toluene was used, both yield and er of **2a** was further decreased (Table 1, entry 4). Although hexane showed better enantioselectivity for this reaction and DMF improved the efficiency of this transformation, both of them failed to give satisfactory results (Table 1, entries 5–6). Yield could be significantly increased with the use of THF or MeCN as solvents, while decreased enantioselectivity was obtained (Table 1, entries 8–9). Subsequently, the examination of palladium salts suggested that Pd[(allyl)Cl]_2_ was the optimal choice, allowing the formation of **2a** in 77% yield with 90:10 er (Table 1, entries 10–13). We then examined the process at 80 °C and observed favorable effects of lower temperature on enantioselectivity (Table 1, entry 14). Increasing the catalyst loading to 10 mol% did not affect the enantioselectivity, but displayed a strong influence on yield (Table 1, entry 15). After a survey of mixed solvents (Table 1, entries 16–20), we were glad to find that a mixed solvent system (Et_2_O/Hexane = 1:1) gave rise to the best result for enantioselectivity (95:5 er) (Table 1, entry 18).

**Table 1:** Optimization of reaction conditions.

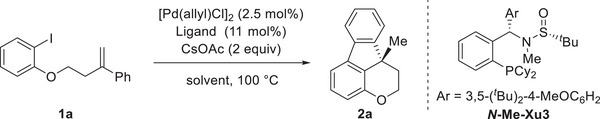			
Entry^a)^	(Pd)	solvent	yield [%]^b)^, ^c)^ (er)
1	Pd_2_(dab)_3_	MTBE	41(89.5:10.5)
2	Pd_2_(dab)_3_	Et_2_O	37(90:10)
3	Pd_2_(dab)_3_	*^i^* Pr_2_O	48(89:11)
4	Pd_2_(dab)_3_	toluene	28(83.5:16.5)
5	Pd_2_(dab)_3_	Hexane	18(91.5:8.5)
6	Pd_2_(dab)_3_	DMF	80(75:25)
7	Pd_2_(dab)_3_	Cyclohexane	35(66.5:33.5)
8	Pd_2_(dab)_3_	THF	93(58:42)
9	Pd_2_(dab)_3_	MeCN	95(72:28)
10	Pd_2_(dab)_3_•CHCl_3_	MTBE	63(90.5:9.5)
11	Pd(OAc)_2_	MTBE	38(86.5:13.5)
12	Pd(TFA)_2_	MTBE	38(87:13)
13	Pd[(allyl)Cl]_2_	MTBE	77(90:10)
14^d)^	Pd[(allyl)Cl]_2_	MTBE	51(93.5:6.5)
15^d)^, ^e)^	Pd[(allyl)Cl]_2_	MTBE	86(93.5:6.5)
16^d)^, ^e)^	Pd[(allyl)Cl]_2_	MTBE/Hexane = 1:1	76(95:5)
17^d)^, ^e)^	Pd[(allyl)Cl]_2_	*^i^* Pr_2_O/Hexane = 1:1	74(94.5:5.5)
18^d)^, ^e)^	Pd[(allyl)Cl]_2_	Et_2_O/Hexane = 1:1	79(95:5)
19^d)^, ^e)^	Pd[(allyl)Cl]_2_	Cyclohexane/MeCN = 1:1	98(75:25)
20^d)^, ^e)^	Pd[(allyl)Cl]_2_	THF/MeCN = 1:1	99(70:30)

^a)^ Unless otherwise noted. All reactions were carried out with 0.1 mmol of **1a**, 5 mol% of catalyst ([Pd] to L = 1:2.2) in 2.0 mL solvent at 100 °C for 24 h;

^b)^ yield with CH_2_Br_2_ as an internal standard was determined by NMR;

^c)^ Determined by chiral HPLC;

^d)^ at 80 °C;

^e)^ 10 mol% of catalyst ([Pd] to L = 1:2.2) was used.

**Figure 2:**
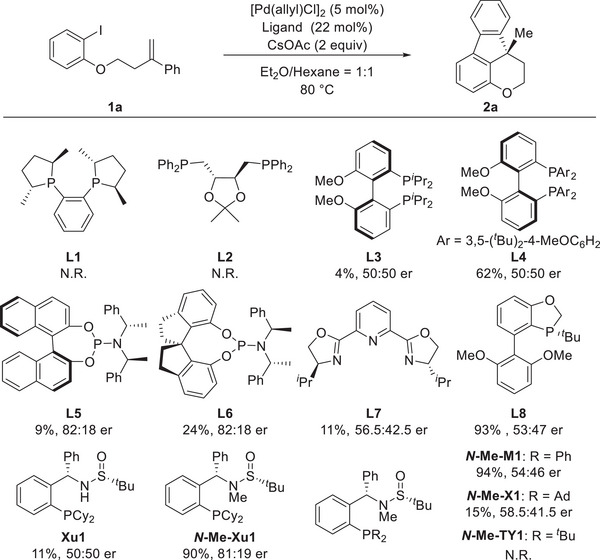
The effect of different ligands in the model reaction.

Furthermore, an exhaustive screening of various types of other commercially available ligands **L1**‐**L8** for model tandem reaction resulted in the formation of the desired product without satisfactory results (**Figure** **2**). The use of other series of Sadphos developed by our group also failed to deliver better results. Accordingly, the optimal conditions in terms of the yield and er thus involve Pd[(allyl)Cl]_2_ as well as ***N*‐Me‐Xu3** with CsOAc in Et_2_O/hexane at 80 °C. However, when we examined the corresponding substrate scope of this reaction, most of the reactions were not complete within 24 h. For example, *tert*‐butyl‐substituted substrate **1b** endured to deliver the desired product after extending the reaction time to 5 days. Thus, in order to improve the efficiency of this transformation, we turned our attention to testing different additives in the aformentioned optimal conditions using **1b** as a model substrate. To our delight, when 50 moL% of AgCl was added to the reaction mixture, product **2b** was efficiently synthesized in 88% isolated yield with 97:3 er within 20 h. (more details in Supporting Information). Noteworthily, AgCl (similar to tetrabutyl ammonium chloride) could shorten the reaction time and minimize side reactions.^[^ ^30^ ^]^

**Figure 3:**
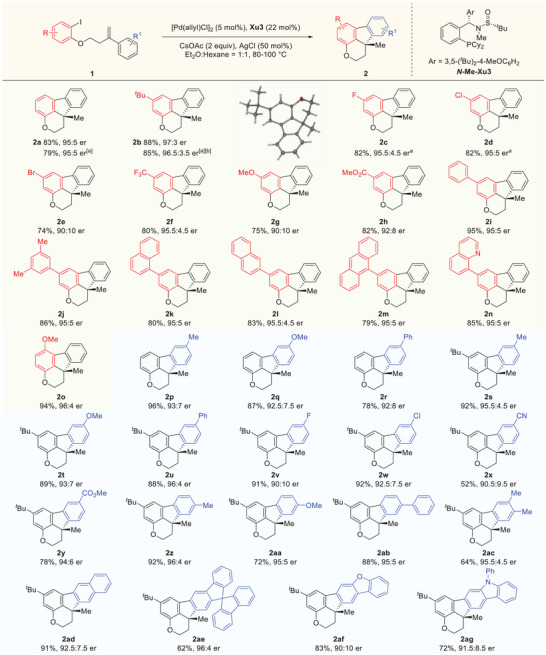
Substrate scope. Conditions: Unless otherwise noted, all reactions were performed with **1** (0.3 mmol), AgCl (50 mol%), CsOAc (0.6 mmol), Pd[(allyl)Cl]_2_ (5 mol%) and ***N*‐Me‐Xu3** (22 mol%)in Et_2_O/Hexane (1:1, 6 mL) at 80–100 °C for 24–48 h. [a] Without AgCl. [b] Run for 5 days.

Having developed the optimum conditions, we first examined the substrate scope with respect to the structural variants on the *ortho*‐iodophenol (**Figure** **3**). Various C4 or C5‐substituted dihydroindeno[1,2,3‐*de*]chromene were efficiently synthesized with the corresponding *ortho*‐iodophenol derivatives regardless of the electronic properties of the substrates. For example, halogenated substituents were well tolerated in this reaction to afford the corresponding products (**2c**‐**2e**) in 74–82% yields with 90:10–95.5:4.5 ers, leaving the halogens available for downstream synthetic manipulation. Substrates bearing functional groups such as ether(**1g**), ester group (**1h**) were also compatible with the reaction conditions, providing the desired products with good enantioselectivities. Furthermore, various aryl and heteroaryl substituents on the aromatic ring of *ortho*‐iodophenol, including phenyl (**1i**), 3, 5‐dimethylphenyl (**1j**), 1‐naphthalene (**1k**), 2‐naphthalene (**1l**), 9‐phenanthrene (**1m**) and 8‐quinoline (**1n**) examples, appeared to have limited effects on the results, and the corresponding products were obtained with excellent results. Additionally, the absolute configuration of **2b** was confirmed by X‐ray diffraction analysis and those of other products were assigned by analogy.^[^ ^31^ ^]^

We next investigated the compatibility of our reaction protocol with a variation of *ortho*‐iodophenol‐derived allyl ether. As shown in Figure 3, it was apparent that the positions and electronic properties of the substituents on the aromatic ring of the allyl ether did not have a significant effect on the stereoselectivity of the process, only compound **2v** and **2x** bearing fluorine group and strongly electron‐withdrawing group (CN) respectively were obtained in slight decrease in enantioselectivities. Notably, on account of steric hindrance effect, products **2z**‐**2ac** were generated with excellent regioselectivity. It should be pointed out that substrate **1ae** derived from 9,9′‐spirobi[fluorene] also reacted smoothly to furnish the desired product in moderate yield with excellent enantioselectivity. Moreover, replacing the phenyl with heteroaryl groups such as dibenzo[*b,d*]furan (**1af**) and 9‐phenyl‐9*H*‐carbazole (**1ag**) produced the corresponding products with good enantioselectivities.

**Figure 4:**
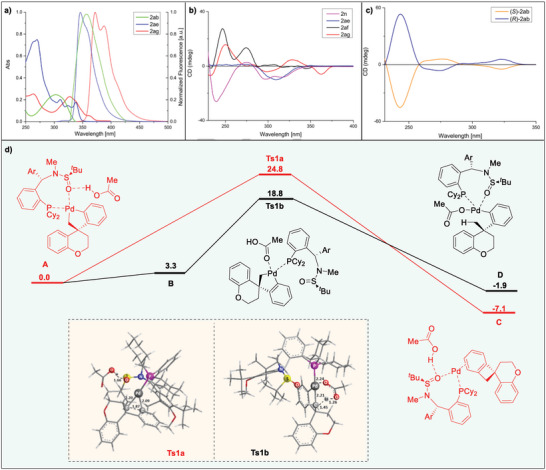
Chiroptical properties and DFT calculations. a) Absorption (solid trace) and emission (dotted trace) spectra of **2ab** (green traces), **2ae** (blue traces), and **2ag** (red traces) recorded in CH_2_Cl_2_ (10^−5^ m) at 25 °C. b) Circular dichroism spectra of **2n** (purple trace), **2ae** (blue trace), **2af** (black trace), **2ag** (red trace) recorded in DCM at 25 °C. c) CD spectra of (S)−**2ab** (navy tarce) and (R)−**2ab** (orange tarce) recorded in DCM at 25 °C. d) Free‐energy reaction profiles (kcal mol^−1^) for two reaction pathways of five‐membered palladacycle intermediate, calculated at the SMD (Et_2_O/Hexane = 1:1) PBE0/combined basis set level at 353 K and the optimized structures of **Ts1a** and **Ts1b**.

Due to the large conjugated systems of fluorene derivatives, these compounds exhibit many unique photoelectric properties and biological activities, and have potential wide applications in photoelectric materials, medicine, and other fields. For example, it can be used as organic light‐emitting diodes,^[^ ^32^ ^]^ solar cell materials,^[^ ^33^, ^34^ ^]^ and even as a biosensor.^[^ ^35^, ^36^, ^37^, ^38^, ^39^ ^]^ Therefore, we conducted optical tests on some of our extended fluorene derivatives. The absorption and emission spectra of selected compounds **2ab**, **2ae** and **2ag** were obtained and are shown in **Figure** **4**a. For comparison, the absorption and emission spectra of other compounds were also measured, their absorption and emission spectral data and quantum efficiency were summarized in **Table** **2** (more details in supporting information). It was worth noting that the maximum absorption and emission wavelengths of the compounds showed significant red shifts, when the phenyl substituents on the allyl aryl groups were in the *p*‐position and *m*‐position, respectively, and the fluorescence quantum efficiency increased from 24% to 85%. For instance, the absorption of the solution of **2ag** at 327 nm was red‐shifted by 54 nm, by comparing with those (273 nm) of the solution of **2a**, and the emission was red‐shifted by 33 nm. Obviously, the change of molecular structure affects the quantum efficiency of the product, and **2ab** was strongly emissive in 1,2‐dichloroethane with quantum efficiency up to 97%. The extremely high fluorescence quantum efficiency proved that these chiral compounds had great potential in the application of fluorescent materials. In order to further examine the configuration and conformation of the chiral compounds, the circular dichroism spectra for compounds **2n**, **2ae**, **2af**, **2ag**, (*S*)−**2ab** and (*R*)−**2ab** were obtained (Figure 4b,c). The different molar ellipticity values observed for compound **2ae** in DCM seem to correlate with the structure of spiral difluorene contained in the molecule. The circular dichroism (CD) analysis further confirmed that the CD peak patterns of the (*S*)−**2ab** and (*R*)−**2ab** mirrored each other in both form and intensity, which was consistent with an enantiomeric relationship.

**Table 2:** Absorption and emission data of compounds.

Compound	λ_abs_ ^a)^/[nm] (ε^b)^ [m ^−1^cm^−1^])	λ_em_/[nm]	*Φ*_F_ ^c)^/[%]
**2a**	273 (17 700)	339	15
**2b**	275 (18 000)	339	17
**2g**	276 (16 100)	349	27
**2n**	296 (46 500)	358	48
**2o**	273 (14 600)	371	20
**2u**	258 (31 400)	336	24
**2ab**	301 (24 600)	356	85(97)^d)^
**2ae**	270 (74 900)	346	68
**2af**	338 (40 400)	351	64
**2ag**	327 (22 800)	372	60

^a)^ Absorption maxima in CH_2_Cl_2_ (10^−5^ m);

^b)^ Molar extinction coefficient;

^c)^ Fluorescence quantum efficiency in CH_2_Cl_2_;

^d)^ Fluorescence quantum efficiency in 1, 2‐dichloroethane.

Five‐membered palladacycle intermediate could undergo the reductive elimination process to furnish spirocycle product. Alternatively, the protonation of palladacycle with the assistance of acetic acid could give rise to arylpalladium complex, which was the result of 1, 4‐Pd shift. To gain deep insight into the two reaction pathways, we performed DFT calculations at PBE0/6‐31G(d)‐LANL2DZ level of theory using Gaussian 09 software package^[^ ^40^ ^]^ and located the transition states leading to both products (see the Supporting Information for details). Figure 4d shows the results of DFT calculations based on the reaction of prototypical **1a** with the Pd(II)−***N*‐Me‐Xu3** chiral system. According to our calculation, the structure of intermediate **A** is more stable when Pd is coordinated with P and O atoms of ligand ***N*‐Me‐Xu3**, in which a strong H‐bonding interaction is found between hydrogen atom of HOAc and the O atom connected with the S atom (1.66 Å). On one hand, the reductive elimination of intermediate **A** proceeds via **Ts1a**, which requires an activation energy of 24.8 kcal mol^−1^, thus forming intermediate **C**, and the spirocycle product is obtained after the subsequent dissociation of complex **C**. On the other hand, intermediate **B**, in which the coordination of the O atom of ligand to the Pd is replaced by the O atom of HOAc in order to facilitate proton transfer process, is generated via the comformational change of intermediate **A**. The proton transfer process needs to overcome a free‐energy barrier of 18.8 kcal mol^−1^ (**Ts1b**) to produce intermediate **D**, in which OAc^−^ is coordinated with Pd in the *k* ^1^ mode.^[^ ^41^ ^]^ These calculations indicate that the energy difference governing the divergence of these reactions is even up to 6.0 kcal mol^−1^, which guarantees the generation of high chemoselectivity of this reaction.

**Figure 5:**
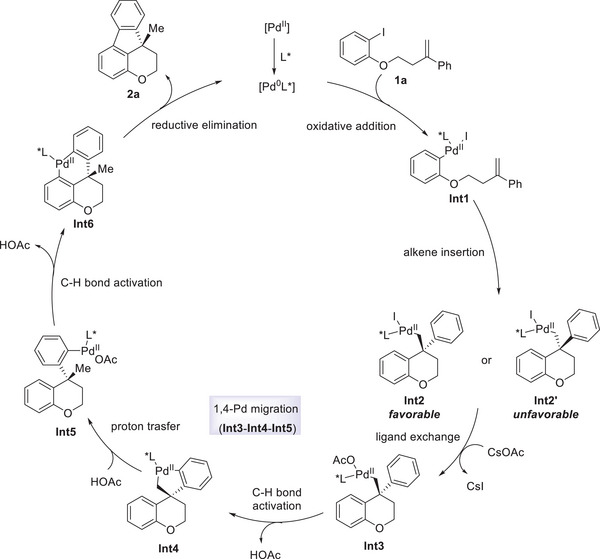
Proposed mechanism.

Moreover, a proposed catalytic mechanism was described in **Figure** **5**. The oxidative addition of **1a** to Pd(0) complex generated intermediate **Int1**, followed by alkene insertion to frunish the favorable complex **Int2**. Then, the ligand exchange of **Int2** with CsOAc gave rise to the formation of **Int3**, which would undrego a concerted metalation−deprotonation pathway to give five‐membered palladacycle **Int4** with the release of HOAc. Subsequent proton transfer was realized with the assistance of HOAc, affording complex **Int5**. The sequence **Int3** → **Int4** → **Int5** resulted in an overall 1,4‐Pd shift. Finally, the second C−H bond activation and the following reductive elimination were occurred to provide product **2a**.

## 3 Conclusion

We have established a robust strategy for the practical and versatile enantioconvergent remote C─H bond activation via carbopalladation and 1,4‐Pd migration—by combining a palladium catalyst with sulfinamide monophosphine ligand ***N*‐Me‐Xu3**, providing a tool to generate enantioenriched fluorene derivatives bearing a quaternary chiral center. This newly developed protocol features a broad substrate scope, wide functional group compatibility, scalable synthetic applications, simple operation as well as high enantioselectivity (up to 97:3 er). The chiral product has a fluorescence quantum yield of up to 97%, indicating the extraordinary promise of this asymmetric catalytic method. Furthermore, DFT calculations provide insights into the origins of the high chemoselectivity of this reaction.

## 4 Experimental Section

To a sealed tube was added Pd[(allyl)Cl]_2_ (5 mol%), ***N***‐**Me**‐**Xu3** (22 mol%), CsOAc (0.6 mmol), AgCl (50 mol%). The flask was evacuated and refilled with argon. Then *o*‐iodophenol‐derived allyl ether **1** (0.3 mmol), a mixed solution of Et_2_O/Hexane (1:1, 6 mL) was added to the tube, and stirred at room temperature for 1 h. Next, the mixture was stirred at 80 °C for 24 h and at 100 °C for another 24 h. After the reaction was complete (monitored by TLC), solvent was removed under reduced pressure. The crude product was then purified by flash column chromatography on silica gel to afford the desired product 2.

## Conflict of Interest

The authors declare no conflict of interest.

## Supporting information

Supporting Information

## Data Availability

The data that support the findings of this study are available in the supplementary material of this article.
