# Supplementary material for: Remote C─H Bond Activation via Enantioselective Carbopalladation and 1,4‐Pd Migration Cascade Process
Source: Adv Sci (Weinh). 2024 Sep 3;11(40):2406443. doi: 10.1002/advs.202406443 (PMC11516156; doi:10.1002/advs.202406443)
Supplement: Supplementary file 1 — Supporting Information [file ADVS-11-2406443-s001.docx]

*Supporting Information*

**Remote C-H bond Activation via Enantioselective Carbopalladation and 1,4-Pd Migration Cascade Process**

Bing Xu,^1,3‡^ Danting Ji,^2‡^ Zhan-Ming Zhang^1,4^* & Junliang Zhang^1,2,5^*

^1^Department of Chemistry, Fudan University, Shanghai, 200438, P.R.China

^2^Shanghai Key Laboratory of Green Chemistry and Chemical Processes, School of Chemistry and Molecular Engineering, East China Normal University, 3663 N. Zhongshan Road, Shanghai 200062, China

^3^Zhuhai Fudan Innovation Institute, Zhuhai, Guangdong, 519000, P.R.China

^4^Fudan Zhangjiang Institute, Shanghai, 201203, P.R.China

^5^School of Chemistry and Chemical Engineering, Henan Normal University, Xinxiang, Henan, 453007, P.R.China

*Corresponding Authors: zhanmingzhang@fudan.edu.cn; junliangzhang@fudan.edu.cn; ^‡^B. Xu and D. Ji contributed equally to this work.

**Contents**

- 1. [General Information S1](#_Toc357886346)
  2. [Table S1. Screening of Bases for Enantioselectiv Remote C-H bond Activation via Carbopalladation and 1, 4-Pd Migration Cascade Process S2](#_Toc357886346)
  3. [Table S2. Screening of Additives for Enantioselectiv Remote C-H bond Activation via Carbopalladation and 1, 4-Pd Migration Cascade Process S2](#_Toc357886346)
  4. [Table S3. Exploring the Effect of AgCl on the Enantioselectiv Remote C-H bond Activation via Carbopalladation and 1, 4-Pd Migration Cascade Process S](#_Toc357886349)3
  5. [General Procedure for the Synthesis of products **2a-2ag** S](#_Toc357886349)3
  6. [Additional Information Regarding the Photophysical of **2a**, **2b**, **2g**, **2n**, **2o**, **2u**, **2ab**, **2af** S](#_Toc357886349)28
  7. [Computational Detailes S](#_Toc357886349)32
  8. [^1^H , ^19^F, ^13^C NMR, HPLC Spectra S](#_Toc357886349)49

1. [References S](#_Toc357886349)117

**1．General Information**

All reactions were carried out under an atmosphere of argon in sealed tube with magnetic stirring. ^1^H NMR spectra of **2a**, **2b**, **2e**, **2i**, **2j**, **2t**, **2v**, **2y**, **2z** and **2aa**-**2ag** were recorded on a Bruker 300 MHz spectrometer in CDCl_3_. The other ^1^H NMR spectra, ^19^F NMR spectra, ^13^C NMR spectra were recorded on a Bruker 400 MHz spectrometer in CDCl_3_. All signals are reported in ppm with the internal TMS signal at 0 ppm as a standard. Data for ^1^H NMR spectra are reported as follows: chemical shift (ppm, referenced to TMS; s = singlet, d = doublet, t = triplet, dd = doublet of doublets, m = multiplet), coupling constant (Hz), and intergration. Data for ^13^C NMR are reported in terms of chemical shift (ppm) relative to residual solvent peak (CDCl_3_: 77.0 ppm). Reactions were monitored by thin layer chromatography (TLC) using silica gel plates. Flash column chromatography was performed over silica gel (300-400 mesh). The substrates **1a-1ag**,^1-2^ were synthesized according to published procedures. The spectral data of the substrates were consisted with that reported in the literature. The enantionmeric excesses of the products were determined by chiral stationary phase HPLC using a Chiralpak IB, IC, ODH.

1. **Table S1. Screening of Bases for Enantioselectiv Remote C-H bond Activation via Carbopalladation and 1, 4-Pd Migration Cascade Process** *^a^*

| Entry | Base | Yield/% ^[b]^ | Er ^[c]^ |
| --- | --- | --- | --- |
| 1 | *^t^*BuOLi | NR | - |
| 2 | *^t^*BuONa | NR | - |
| 3 | K_2_CO_3_ | NR | - |
| 4 | Cs_2_CO_3_ | NR | - |
| 5 | Na_2_CO_3_ | NR | - |
| 6 | KOH | NR | - |
| 7 | NaOH | NR | - |
| 8 | CsOAc | 41 | 89.5:10.5 |
| [a] Unless otherwise noted. All reactions were carried out with 0.1 mmol of **1a**, 10 mol% of catalyst ([Pd] to **L** = 1:2.2), 0.2 mmol Base in 2 mL MTBE at 100 ºC for 24 h. [b] Conversion and NMR yield with CH_2_Br_2_ as an internal standard. [c] Determined by chiral HPLC. | | | |

1. **Table S2. Screening of Additives for Enantioselectiv Remote C-H bond Activation via Carbopalladation and 1, 4-Pd Migration Cascade Process** *^a^*

| Entry | Additive | Conv./%^[b]]^ | Yield/% ^[b^ | Er ^[c]^ |
| --- | --- | --- | --- | --- |
| 1 | AgOAc | 78 | 63 | 90:10 |
| 2 | Ag_2_CO_3_ | 36 | 10 | 71.5:28.5 |
| 3 | AgF | 100 | 81 | 97:3 |
| 4 | AgCl | 100 | 88 | 97:3 |
| 5 | TBAB | 100 | 80 | 96.5:3.5 |
| 6 | TBAC | 100 | 82 | 97:3 |
| 7 | 3Å MS | 83 | 73 | 97:3 |
| 8 | 4Å MS | 37 | 27 | 96.5:3.5 |
| 9 | 5Å MS | 83 | 68 | 97.5:2.5 |
| [a] Unless otherwise noted. All reactions were carried out with 0.1 mmol of **1b**, 10 mol% of catalyst ([Pd] to **L** = 1:2.2), 0.2 mmol Cs_2_CO_3_, additive (50 mol%) or molecular sieve (30 mg) in 2 mL Et_2_O/Hexane at 80 ºC for 20 h. [b] Conversion and NMR yield with CH_2_Br_2_ as an internal standard. [c] Determined by chiral HPLC. | | | | |

1. **Table S3. Exploring the Effect of AgCl on the Enantioselectiv Remote C-H bond Activation via Carbopalladation and 1, 4-Pd Migration Cascade Process** *^a^*

| Entry | x | t/h | Conv./%^[b]]^ | Yield/% ^[b^ | *Ee*/% ^[c]^ |
| --- | --- | --- | --- | --- | --- |
| 1 | 5 | 60 | 67 | 45 | 96.5:3.5 |
| 2 | 10 | 60 | 70 | 50 | 96:4 |
| 3 | 15 | 60 | 54 | 40 | 96:4 |
| 4 | 20 | 60 | 93 | 78 | 95.5:4.5 |
| 5 | 30 | 48 | 86 | 72 | 95.5:4.5 |
| 6 | 50 | 20 | 100 | 88 | 97:3 |
| 7 | 100 | 8 | 100 | 83 | 91.5:8.5 |
| [a] Unless otherwise noted. All reactions were carried out with 0.1 mmol of **1b**, 10 mol% of catalyst ([Pd] to **L** = 1:2.2), 0.2 mmol Cs_2_CO_3_, Ag (x mol%) in 2 mL Et_2_O/Hexane at 80 ºC for 20 h. [b] Conversion and NMR yield with CH_2_Br_2_ as an internal standard. [c] Determined by chiral HPLC. | | | | | |

1. **General Procedure for the Synthesis of products 2a-2ag**

**Typical procedure A for Enantioselectiv Remote C-H bond Activation via Carbopalladation and 1, 4-Pd Migration Cascade Process**

To a sealed tube was added Pd[(allyl)Cl]_2_ (5 mol%), ***N*-Me-Xu3** (22 mol%), CsOAc (0.6 mmol). The flask was evacuated and refilled with argon. Then *o*-iodophenol-derived allyl ether 1 (0.3 mmol), a mixed solution of Et_2_O/Hexane (1:1, 6 mL) was added to the tube, and stirred at room temperature for 1 h. Next, the mixture was stirred at 80 °C for 24 h. After the reaction was complete (monitored by TLC), solvent was removed under reduced pressure. The crude product was then purified by flash column chromatography on silica gel to afford the desired product.

**Typical procedure B for Enantioselectiv Remote C-H bond Activation via Carbopalladation and 1, 4-Pd Migration Cascade Process**

To a sealed tube was added Pd[(allyl)Cl]_2_ (5 mol%), ***N*-Me-Xu3** (22 mol%), CsOAc (0.6 mmol), AgCl (50 mol%). The flask was evacuated and refilled with argon. Then *o*-iodophenol-derived allyl ether 1 (0.3 mmol), a mixed solution of Et_2_O/Hexane (1:1, 6 mL) was added to the tube, and stirred at room temperature for 1 h. Next, the mixture was stirred at 80 °C for 24 h. After the reaction was complete (monitored by TLC), solvent was removed under reduced pressure. The crude product was then purified by flash column chromatography on silica gel to afford the desired product.

**Typical procedure C for Enantioselectiv Remote C-H bond Activation via Carbopalladation and 1, 4-Pd Migration Cascade Process**

To a sealed tube was added Pd[(allyl)Cl]_2_ (5 mol%), ***N*-Me-Xu3** (22 mol%), CsOAc (0.6 mmol), AgCl (50 mol%). The flask was evacuated and refilled with argon. Then *o*-iodophenol-derived allyl ether 1 (0.3 mmol), a mixed solution of Et_2_O/Hexane (1:1, 6 mL) was added to the tube, and stirred at room temperature for 1 h. Next, the mixture was stirred at 80 °C for 24 h and at 100 °C for another 24 h. After the reaction was complete (monitored by TLC), solvent was removed under reduced pressure. The crude product was then purified by flash column chromatography on silica gel to afford the desired product.

1. Synthesis of (*S*)-10b-methyl-1,10b-dihydro-2*H*-indeno[1,2,3-*de*]chromene (**2a)**.

Prepared according to typical procedure **A** from allyl ether **1a** (105.0 mg, 0.3 mmol), after a flash column chromatography (hexanes: EA = 200:1) afforded the product **2a** as a colorless liquid (52.6 mg, 79% yield) with 95:5 er. According to typical procedure **B**, **2a** was obtained in 83% yield with 95:5 er. ^1^H NMR (300 MHz, CDCl_3_) δ 7.68 (dt, *J* = 7.6, 0.9 Hz, 1 H), 7.41 (dt, *J* = 7.2, 1.0 Hz, 1 H), 7.32 (td, *J* = 7.4, 1.4 Hz, 1 H), 7.26 (dd, *J* = 7.3, 1.3 Hz, 1 H), 7.22 (dd, *J* = 4.4, 0.9 Hz, 2 H), 6.71 (p, *J* = 3.9 Hz, 1 H), 4.62 (ddd, *J* = 13.0, 11.5, 4.0 Hz, 1 H), 4.53 (ddd, *J* = 11.4, 6.3, 1.2 Hz, 1 H), 2.28 (ddd, *J* = 13.0, 4.0, 1.2 Hz, 1 H), 1.63 (td, *J* = 13.0, 6.3 Hz, 1 H), 1.44 (s, 3 H). ^13^C NMR (400 MHz, CDCl_3_) δ 153.67, 152.78, 140.42, 140.15, 134.67, 129.29, 127.31, 126.88, 122.97, 121.04, 113.53, 112.32, 65.48, 42.43, 30.86, 25.48. MS (EI): m/z (%) = 222 (M^+^, 47.70), 207 (100)；HRMS calculated for [C_16_H_14_O]^+^: 222.1045 found: 222.1048. Enantiomeric excess was determined by HPLC with a Chiralpak IC column (hexanes: 2-propanol = 99:1, 0. 5 mL/min, 254 nm); minor enantiomer tr = 10.3 min, major enantiomer tr = 9.5 min. [α]_D_^20^ = -76.5 (*c* = 0.4, CHCl_3_).

1. Synthesis of (*S*)-5-(tert-butyl)-10b-methyl-1,10b-dihydro-2*H*-indeno[1,2,3-*de*] chromene (**2b)**.

Prepared according to typical procedure **B** from allyl ether **1b** (121.9 mg, 0.3 mmol), after a flash column chromatography (hexanes: Et_2_O = 100:1) afforded the product **2b** as a white solid (73.5 mg, 88% yield) with 97:3 er. According to typical procedure **A** and extending the reaction time to 5 days, **2b** was obtained in 85% yield with 96.5:3.5 er. Mp: 111-112 ^o^C. ^1^H NMR (300 MHz, CDCl_3_) δ 7.72-7.69 (m, 1 H), 7.40 (ddd, *J* = 7.3, 1.3, 0.7 Hz, 1 H), 7.35-7.29 (m, 2 H), 7.24 (td, *J* = 7.3, 1.2 Hz, 1 H), 6.77 (d, *J* = 1.4 Hz, 1 H), 4.63 (ddd, *J* = 13.0, 11.4, 3.8 Hz, 1 H), 4.53 (ddd, *J* = 11.5, 6.2, 1.2 Hz, 1 H), 2.26 (ddd, *J* = 13.0, 3.8, 1.3 Hz, 1 H), 1.64 (td, *J* = 13.0, 6.2 Hz, 1 H), 1.44 (s, 3 H), 1.36 (s, 9 H). ^13^C NMR (400 MHz, CDCl_3_) δ 154.10, 153.50, 152.37, 140.42, 140.14, 131.87, 127.19, 126.64, 122.96, 120.81, 110.75, 109.37, 65.45, 42.10, 35.06, 31.71, 31.05, 25.63. MS (EI): m/z (%) = 278 (M^+^, 38.07), 263 (100)；HRMS calculated for [C_20_H_22_O]^+^: 278.1671 found: 278.1669. Enantiomeric excess was determined by HPLC with a Chiralpak ODH column (hexanes: 2-propanol = 99:1, 0.5 mL/min, 254 nm); minor enantiomer tr = 8.3 min, major enantiomer tr = 8.7 min. [α]_D_^20^ = -81.7 (*c* = 0.4, CHCl_3_).

1. Synthesis of (*S*)-5-fluoro-10b-methyl-1,10b-dihydro-2*H*-indeno[1,2,3-*de*] chromene (**2c**).

Prepared according to typical procedure **A** from allyl ether **1c** (110.5 mg, 0.3 mmol), after a flash column chromatography (hexanes: EA = 100:1) afforded the product **2c** as a colorless liquid (59.2 mg, 82% yield) with 95.5:4.5 er. ^1^H NMR (400 MHz, CDCl_3_) δ 7.64-7.62 (m, 1 H), 7.42-7.40 (m, 1 H), 7.31 (dtd, *J* = 21.2, 7.4, 1.3 Hz, 2 H), 6.93 (dd, *J* = 8.6, 2.0 Hz, 1 H), 6.44 (dd, *J* = 10.6, 2.0 Hz, 1 H), 4.61 (ddd, *J* = 13.1, 11.5, 3.9 Hz, 1 H), 4.53 (ddd, *J* = 11.5, 6.2, 1.2 Hz, 1 H), 2.27 (ddd, *J* = 13.1, 3.9, 1.1 Hz, 1 H), 1.60 (td, *J* = 13.2, 6.3 Hz, 1 H), 1.42 (s, 3 H). ^19^F NMR (400 MHz, CDCl_3_) δ -112.98. ^13^C NMR (400 MHz, CDCl_3_) δ 164.37 (d, *J* = 242.8 Hz), 154.06, 153.07 (d, *J* = 13.9 Hz), 140.93 (d, *J* = 11.2 Hz), 139.29 (d, *J* = 3.7 Hz), 130.17 (d, *J* = 2.2 Hz), 127.42, 123.00, 121.16, 100.98 (d, *J* = 26.7 Hz), 99.72 (d, *J* = 24.4 Hz), 65.83, 42.09, 30.75, 25.50. MS (EI): m/z (%) = 240 (M^+^, 37.31), 225 (100)；HRMS calculated for [C_16_H_13_OF]^+^: 240.0950 found: 240.0954. Enantiomeric excess was determined by HPLC with a Chiralpak ODH+ODH column (hexanes: 2-propanol = 99:1, 0.5 mL/min, 254 nm); minor enantiomer tr = 24.6 min, major enantiomer tr = 26.0 min. [α]_D_^20^ = -59.7 (*c* = 0.4, CHCl_3_).

1. Synthesis of (*S*)-5-chloro-10b-methyl-1,10b-dihydro-2*H*-indeno[1,2,3-*de*] chromene (**2d)**.

Prepared according to typical procedure **A** from allyl ether **1d** (115.4 mg, 0.3 mmol), after a flash column chromatography (hexanes: EA = 100:1) afforded the product **2d** as a colorless liquid (63.3 mg, 82% yield) with 95:5 er. ^1^H NMR (400 MHz, CDCl_3_) δ 7.64-7.62 (m, 1 H), 7.41-7.39 (m, 1 H), 7.34-7.31 (m, 1 H), 7.28 (td, *J* = 7.3, 1.3 Hz, 1 H), 7.20 (d, *J* = 1.5 Hz, 1 H), 6.72 (d, *J* = 1.6 Hz, 1 H), 4.60 (ddd, *J* = 13.1, 11.5, 3.9 Hz, 1 H), 4.52 (ddd, *J* = 11.5, 6.2, 1.2 Hz, 1 H), 2.27 (ddd, *J* = 13.0, 3.9, 1.2 Hz, 1 H), 1.59 (td, *J* = 13.1, 6.4 Hz, 1 H), 1.40 (s, 3 H). ^13^C NMR (400 MHz, CDCl_3_) δ 153.72, 153.03, 141.26, 138.97, 134.35, 133.08, 127.53, 127.47, 123.05, 121.19, 113.74, 112.76, 65.79, 42.22, 30.64, 25.33. MS (EI): m/z (%) = 256 (M^+^, 39.82), 241 (100)；HRMS calculated for [C_16_H_13_OCl]^+^: 256.0655 found: 256.0657. Enantiomeric excess was determined by HPLC with a Chiralpak ODH+ODH column (hexanes: 2-propanol = 99:1, 0.5 mL/min, 254 nm); minor enantiomer tr = 25.0 min, major enantiomer tr = 26.4 min. [α]_D_^20^ = -86.0 (*c* = 0.4, CHCl_3_).

1. Synthesis of (*S*)-5-bromo-10b-methyl-1,10b-dihydro-2*H*-indeno[1,2,3-*de*] chromene (**2e)**.

Prepared according to typical procedure **B** from allyl ether **1e** (128.7 mg, 0.3 mmol), after a flash column chromatography (hexanes: Et_2_O = 100:1) afforded the product **2e** as a colorless liquid (66.6 mg, 74% yield) with 90:10 er. ^1^H NMR (300 MHz, CDCl_3_) δ 7.66-7.63 (m, 1 H), 7.42 (dd, *J* = 7.4, 1.3 Hz, 1 H), 7.37-7.26 (m, 3 H), 6.88 (d, *J* = 1.4 Hz, 1 H), 4.67-4.51(m, 2 H), 2.29 (ddd, *J* = 13.1, 3.9, 1.3 Hz, 1 H), 1.62 (dt, *J* = 13.2, 6.6 Hz, 1 H), 1.42 (s, 3 H). ^13^C NMR (400 MHz, CDCl_3_) δ 153.63, 153.30, 141.69, 138.89, 133.60, 127.57, 127.51, 123.07, 122.03, 121.24, 116.58, 115.64, 65.84, 42.30, 30.62, 25.28. MS (EI): m/z (%) = 300 (M^+^, 43.80), 285 (100)；HRMS calculated for [C_16_H_13_OBr]^+^: 300.0150 found: 300.0153. Enantiomeric excess was determined by HPLC with a Chiralpak ODH+ODH column (hexanes: 2-propanol = 99:1, 0.5 mL/min, 254 nm); minor enantiomer tr = 26.2 min, major enantiomer tr = 27.7 min. [α]_D_^20^ = -93.7 (*c* = 0.4, CHCl_3_).

1. Synthesis of (*S*)-10b-methyl-5-(trifluoromethyl)-1,10b-dihydro-2*H*-indeno [1,2,3-*de*]chromene (**2f)**.

Prepared according to typical procedure **B** from allyl ether **1f** (125.5 mg, 0.3 mmol), after a flash column chromatography (hexanes: EA = 100:1) afforded the product **2f** as a green liquid (69.9 mg, 80% yield) with 95.5:4.5 er. ^1^H NMR (400 MHz, CDCl_3_) δ 7.73 (dt, *J* = 7.6, 0.9 Hz, 1 H), 7.48-7.44 (m, 2 H), 7.38 (td, *J* = 7.4, 1.4 Hz, 1 H), 7.32 (td, *J* = 7.4, 1.4 Hz, 1 H), 6.98 (s, 1 H), 4.67 (ddd, *J* = 13.1, 11.5, 4.0 Hz, 1 H), 4.59 (ddd, *J* = 11.6, 6.3, 1.2 Hz, 1 H), 2.35 (ddd, *J* = 13.1, 4.0, 1.2 Hz, 1 H), 1.65 (td, *J* = 13.1, 6.3 Hz, 1 H), 1.45 (s, 3 H). ^19^F NMR (400 MHz, CDCl_3_) δ -61.80. ^13^C NMR (400 MHz, CDCl_3_) δ 153.60, 152.68, 140.96, 138.96, 137.92, 132.09, 131.78, 127.73 (d, *J* = 13.5 Hz), 124.36 (q, *J* = 271.0 Hz), 123.15, 121.38, 110.91 (q, *J* = 4.0 Hz), 109.20-109.12 (m), 65.92, 42.61, 30.49, 25.21. MS (EI): m/z (%) = 290 (M^+^, 47.33), 275 (100)；HRMS calculated for [C_17_H_13_OF_3_]^+^: 290.0918 found: 290.0925. Enantiomeric excess was determined by HPLC with a Chiralpak ODH column (hexanes: 2-propanol = 99:1, 0.5 mL/min, 254 nm); minor enantiomer tr = 10.7 min, major enantiomer tr = 11.2 min. [α]_D_^20^ = -80.9 (*c* = 0.4, CHCl_3_).

1. Synthesis of (*S*)-5-methoxy-10b-methyl-1,10b-dihydro-2*H*-indeno[1,2,3-*de*] chromene (**2g)**.

Prepared according to typical procedure **B** from allyl ether **1g** (114.0 mg, 0.3 mmol), after a flash column chromatography (hexanes: Et_2_O = 100:1) afforded the product **2g** as a colorless liquid (53.5 mg, 75% yield) with 90:10 er. ^1^H NMR (400 MHz, CDCl_3_) δ 7.68 (d, *J* = 7.5 Hz, 1 H), 7.43 (d, *J* = 7.4 Hz, 1 H), 7.35 (td, *J* = 7.4, 1.3 Hz, 1 H), 7.32 – 7.23 (m, 1 H), 6.85 (d, *J* = 1.8 Hz, 1 H), 6.33 (d, *J* = 1.8 Hz, 1 H), 4.65 (ddd, *J* = 13.5, 11.5, 3.8 Hz, 1 H), 4.55 (dd, *J* = 11.5, 6.0 Hz, 1 H), 3.85 (s, 3 H), 2.29 (dd, *J* = 13.0, 3.8 Hz, 1 H), 1.67 (dd, *J* = 13.1, 6.1 Hz, 1 H), 1.46 (s, 3 H). ^13^C NMR (400 MHz, CDCl_3_) δ 161.86, 154.31, 153.22, 140.88, 139.95, 127.24, 126.98, 122.96, 120.93, 99.33, 98.75, 65.65, 55.72, 41.93, 31.10, 25.68. MS (EI): m/z (%) = 252 (M^+^, 31.43), 237 (100)；HRMS calculated for [C_17_H_16_O_2_]^+^: 252.1150 found: 252.1154. Enantiomeric excess was determined by HPLC with a Chiralpak IC column (hexanes: 2-propanol = 99:1, 0.5 mL/min, 254 nm); minor enantiomer tr = 15.7 min, major enantiomer tr = 14.9 min. [α]_D_^20^ = -60.9 (*c* = 0.4, CHCl_3_).

1. Synthesis of (*S*)-10b-methyl-1,10b-dihydro-2*H*-indeno[1,2,3-*de*]chromene-5- carboxylate (**2h)**.

Prepared according to typical procedure **B** from allyl ether **1h** (81.6 mg, 0.2 mmol), after a flash column chromatography (hexanes: EA = 20:1) afforded the product **2h** as a colorless liquid (45.9 mg, 82% yield) with 92:8 er. ^1^H NMR (400 MHz, CDCl_3_) δ 7.93 (d, *J* = 1.1 Hz, 1 H), 7.75 (dt, *J* = 7.5, 0.9 Hz, 1 H), 7.43 (td, *J* = 4.1, 3.5, 1.1 Hz, 2 H), 7.36 (td, *J* = 7.5, 1.3 Hz, 1 H), 7.30 (td, *J* = 7.4, 1.3 Hz, 1 H), 4.65 (ddd, *J* = 13.1, 11.5, 4.0 Hz, 1 H), 4.57 (ddd, *J* = 11.5, 6.3, 1.1 Hz, 1 H), 3.92 (s, 3 H), 2.32 (ddd, *J* = 13.1, 4.1, 1.1 Hz, 1 H), 1.64 (td, *J* = 13.1, 6.2 Hz, 1 H), 1.44 (s, 3 H). ^13^C NMR (400 MHz, CDCl_3_) δ 167.26, 153.40, 152.41, 140.38, 139.47, 139.25, 131.69, 127.55, 127.45, 123.02, 121.31, 115.19, 113.68, 65.64, 52.07, 42.63, 30.50, 25.17. MS (EI): m/z (%) = 280 (M^+^, 53.27), 265 (100)；HRMS calculated for [C_18_H_16_O_3_]^+^: 280.1099 found: 280.1103. Enantiomeric excess was determined by HPLC with a Chiralpak ODH column (hexanes: 2-propanol = 98:2, 0.5 mL/min, 254 nm); minor enantiomer tr = 22.3 min, major enantiomer tr = 27.7 min. [α]_D_^20^ = -104.4 (*c* = 0.4, CHCl_3_).

1. Synthesis of (*S*)-10b-methyl-5-phenyl-1,10b-dihydro-2*H*-indeno[1,2,3-*de*] chromene (**2i)**.

Prepared according to typical procedure **B** from allyl ether **1i** (85.2 mg, 0.2 mmol), after a flash column chromatography (hexanes: Et_2_O = 100:1) afforded the product **2i** as a colorless liquid (54.1 mg, 95% yield) with 95:5 er. ^1^H NMR (300 MHz, CDCl_3_) δ 7.75 (dt, *J* = 7.6, 0.9 Hz, 1 H), 7.64-7.61 (m, 2 H), 7.46-7.41 (m, 4 H), 7.39-7.26 (m, 3 H), 6.95 (d, *J* = 1.3 Hz, 1 H), 4.68 (ddd, *J* = 13.0, 11.4, 3.9 Hz, 1 H), 4.59 (ddd, *J* = 11.5, 6.2, 1.2 Hz, 1 H), 2.33 (ddd, *J* = 13.0, 3.9, 1.2 Hz, 1 H), 1.70 (td, *J* = 13.1, 6.2 Hz, 1 H), 1.49 (s, 3 H). ^13^C NMR (400 MHz, CDCl_3_) δ 153.93, 152.90, 143.33, 141.75, 140.88, 139.98, 133.77, 128.65, 128.64, 127.31, 127.13, 127.05, 123.03, 121.04, 112.61, 111.46, 65.61, 42.30, 30.94, 25.54. MS (EI): m/z (%) = 298 (M+, 46.35), 283 (100)；HRMS calculated for [C_22_H_18_O]+: 298.1358 found: 298.1357. Enantiomeric excess was determined by HPLC with a Chiralpak ODH column (hexanes: 2-propanol = 99:1, 0.5 mL/min, 254 nm); minor enantiomer tr = 33.4 min, major enantiomer tr = 31.4 min. [α]_D_^20^ = -117.6 (*c* = 0.4, CHCl_3_).

1. Synthesis of (*S*)-5-(3,5-dimethylphenyl)-10b-methyl-1,10b-dihydro-2*H*-indeno [1,2,3-*de*]chromene (**2j)**.

Prepared according to typical procedure **B** from allyl ether **1j** (90.8mg, 0.2 mmol), after a flash column chromatography (hexanes: Et_2_O = 100:1) afforded the product **2j** as a colorless liquid (56.0 mg, 86% yield) with 95:5 er. ^1^H NMR (300 MHz, CDCl_3_) δ 7.76-7.73 (m, 1 H), 7.43 (td, *J* = 3.4, 1.2 Hz, 2 H), 7.35 (td, *J* = 7.4, 1.4 Hz, 1 H), 7.29 (dd, *J* = 7.3, 1.3 Hz, 1 H), 7.24 (s, 2 H), 6.98 (s, 1 H), 6.93 (d, *J* = 1.2 Hz, 1 H), 4.67 (ddd, *J* = 13.1, 11.5, 3.9 Hz, 1 H), 4.57 (ddd, *J* = 11.5, 6.3, 1.2 Hz, 1 H), 2.38 (s, 6 H), 2.31 (ddd, *J* = 13.0, 3.9, 1.2 Hz, 1 H), 1.69 (td, *J* = 13.1, 6.2 Hz, 1 H), 1.48 (s, 3 H). ^13^C NMR (400 MHz, CDCl_3_) δ 153.97, 152.83, 143.60, 141.78, 140.76, 140.10, 138.10, 133.64, 128.79, 127.34, 126.97, 125.28, 123.02, 121.05, 112.63, 111.51, 65.59, 42.31, 31.00, 25.55, 21.40. MS (EI): m/z (%) = 326 (M^+^, 42.01), 311 (100)；HRMS calculated for [C_24_H_22_O]^+^: 326.1671 found: 326.1674. Enantiomeric excess was determined by HPLC with a Chiralpak ODH column (hexanes: 2-propanol = 99:1, 0.5 mL/min, 254 nm); minor enantiomer tr = 16.8 min, major enantiomer tr = 14.6 min. [α]_D_^20^ = -99.0 (*c* = 0.4, CHCl_3_).

1. Synthesis of (*S*)-10b-methyl-5-(naphthalen-1-yl)-1,10b-dihydro-2*H*-indeno [1,2,3-*de*]chromene (**2k)**.

Prepared according to typical procedure **B** from allyl ether **1k** (95.2 mg, 0.2 mmol), after a flash column chromatography (hexanes: Et_2_O = 100:1) afforded the product **2k** as a white solid (55.9 mg, 80% yield) with 95:5 er. Mp: 87-88 ^o^C. ^1^H NMR (400 MHz, CDCl_3_) δ 8.08 (d, *J* = 8.4 Hz, 1 H), 7.91 (dd, *J* = 18.5, 7.9 Hz, 2 H), 7.72 (d, *J* = 7.3 Hz, 1 H), 7.58 – 7.45 (m, 5 H), 7.41 – 7.32 (m, 3 H), 6.91 (d, *J* = 1.3 Hz, 1 H), 4.79 – 4.71 (m, 1 H), 4.65 (dd, *J* = 11.5, 6.1 Hz, 1 H), 2.40 (dd, *J* = 13.0, 3.8 Hz, 1 H), 1.81 (td, *J* = 13.1, 6.1 Hz, 1 H), 1.59 (s, 3 H). ^13^C NMR (400 MHz, CDCl_3_) δ 154.03, 152.45, 142.31, 140.64, 140.28, 140.05, 133.77, 133.65, 131.79, 127.50, 127.40, 127.04, 126.77, 126.31, 125.92, 125.70, 125.32, 123.07, 121.11, 115.40, 114.41, 65.63, 42.42, 30.95, 25.67. MS (EI): m/z (%) = 348 (M^+^, 58.67), 333 (100)；HRMS calculated for [C_26_H_20_O]^+^: 348.1514 found: 348.1517. Enantiomeric excess was determined by HPLC with a Chiralpak ODH column (hexanes: 2-propanol = 95:5, 0.5 mL/min, 254 nm); minor enantiomer tr = 25.9 min, major enantiomer tr = 29.7 min. [α]_D_^20^ = -77.7 (*c* = 0.4, CHCl_3_).

1. Synthesis of (*S*)-10b-methyl-5-(naphthalen-2-yl)-1,10b-dihydro-2*H*-indeno [1,2,3-*de*]chromene (**2l)**.

Prepared according to typical procedure **B** from allyl ether **1l** (95.2 mg, 0.2 mmol), after a flash column chromatography (hexanes: Et_2_O = 100:1) afforded the product **2l** as a colorless liquid (57.6 mg, 83% yield) with 95.5:4.5 er. ^1^H NMR (400 MHz, CDCl_3_) δ 8.10 – 8.09 (m, 1 H), 7.94 – 7.82 (m, 3 H), 7.82 – 7.79 (m, 2 H), 7.60 (d, *J* = 1.3 Hz, 1 H), 7.52 – 7.47 (m, 3 H), 7.42 – 7.38 (m, 1 H), 7.34 – 7.30 (td, *J* = 7.4, 1.2 Hz, 1H), 7.10 (d, *J* = 1.3 Hz, 1 H), 4.76 – 7.69 (m, 1 H), 4.66 – 4.61 (m, 1 H), 2.40 – 2.35 (m, 1 H), 1.79 – 1.69 (m, 1 H), 1.54 (s, 3 H). ^13^C NMR (400 MHz, CDCl_3_) δ 153.99, 153.02, 143.23, 141.03, 140.01, 139.12, 133.94, 133.67, 132.63, 128.28, 128.19, 127.62, 127.43, 127.12, 126.22, 125.90, 125.88, 125.82, 123.09, 121.14, 112.90, 111.73, 65.70, 42.38, 31.01, 25.57. MS (EI): m/z (%) = 348 (M^+^, 50.39), 333 (100)；HRMS calculated for [C_26_H_20_O]^+^: 348.1514 found: 348.1515. Enantiomeric excess was determined by HPLC with a Chiralpak ODH column (hexanes: 2-propanol = 99:1, 0.5 mL/min, 254 nm); minor enantiomer tr = 28.4 min, major enantiomer tr = 24.0 min. [α]_D_^20^ = -131.4 (*c* = 0.4, CHCl_3_).

1. Synthesis of (*S*)-5-(anthracen-9-yl)-10b-methyl-1,10b-dihydro-2*H*-indeno [1,2,3-*de*]chromene (**2m)**.

Prepared according to typical procedure **B** from allyl ether **1m** (131.9 mg, 0.25 mmol), after a flash column chromatography (hexanes: Et_2_O = 100:1) afforded the product **2m** as a white solid (78.9 mg, 79% yield) with 95:5 er. Mp: 88-89 ^o^C. ^1^H NMR (400 MHz, CDCl_3_) δ 8.79 (dd, *J* = 20.8, 8.3 Hz, 2 H), 8.13 (d, *J* = 8.2 Hz, 1 H), 7.94 (d, *J* = 7.7 Hz, 1 H), 7.81 (s, 1 H), 7.76 – 7.57 (m, 3 H), 7.65 (t, *J* = 7.4 Hz, 1 H), 7.59 (t, *J* = 7.6 Hz, 1 H), 7.52 (d, *J* = 7.3 Hz, 1 H), 7.47 (s, 1 H), 7.37 (dt, *J* = 18.7, 7.4 Hz, 2 H), 6.99 (s, 1 H), 4.76 (td, *J* = 12.4, 3.8 Hz, 1 H), 4.67 (dd, *J* = 11.5, 6.1 Hz, 1 H), 2.41 (dd, *J* = 13.2, 3.8 Hz, 1 H), 1.83 (td, *J* = 13.1, 6.1 Hz, 1 H), 1.61 (s, 3 H). ^13^C NMR (400 MHz, CDCl_3_) δ 154.01, 152.50, 142.32, 140.33, 140.01, 139.14, 133.79, 131.57, 131.31, 130.57, 129.92, 128.62, 127.40, 127.32, 127.18, 127.06, 126.76, 126.47, 126.45, 126.38, 123.05, 122.79, 122.49, 121.13, 115.40, 114.36, 65.64, 42.44, 30.95, 25.65. MS (EI): m/z (%) = 398 (M^+^, 71.79), 383 (100)；HRMS calculated for [C_30_H_22_O]^+^: 398.1671 found: 398.1678. Enantiomeric excess was determined by HPLC with a Chiralpak ODH column (hexanes: 2-propanol = 90:10, 0.5 mL/min, 254 nm); minor enantiomer tr = 21.0 min, major enantiomer tr = 27.2 min. [α]_D_^20^ = -58.1 (*c* = 0.4, CHCl_3_).

1. Synthesis of (*S*)-8-(10b-methyl-1,10b-dihydro-2*H*-indeno[1,2,3-*de*]chromen- 5-yl)quinoline (**2n)**.

Prepared according to typical procedure **B** from allyl ether **1n** (95.2 mg, 0.2 mmol), after a flash column chromatography (hexanes: EA = 10:1) afforded the product **2n** as a yellow liquid (59.2 mg, 85% yield) with 95:5 er. ^1^H NMR (400 MHz, CDCl_3_) δ 9.00 – 8.99 (m, 1 H), 8.22 (d, *J* = 8.2 Hz, 1 H), 7.81 (dd, *J* = 12.0, 7.6 Hz, 2 H), 7.73 (d, *J* = 7.4 Hz, 1 H), 7.63 – 7.58 (m, 2 H), 7.47 (d, *J* = 7.4 Hz, 1 H), 7.42 (dd, *J* = 8.3, 4.2 Hz, 1 H), 7.35 (t, *J* = 7.4 Hz, 1 H), 7.28 (dd, *J* = 13.3, 6.0 Hz, 1 H), 7.10 (s, 1H), 4.72 (td, *J* = 12.4, 4.0 Hz, 1 H), 4.62 (dd, *J* = 11.4, 6.1 Hz, 1 H), 2.36 (dd, *J* = 13.0, 3.9 Hz, 1 H), 1.78 (td, *J* = 13.0, 6.2 Hz, 1 H), 1.55 (s, 3 H). ^13^C NMR (400 MHz, CDCl_3_) δ 153.94, 152.24, 150.21, 146.07, 141.29, 141.08, 140.36, 140.10, 136.22, 133.74, 130.45, 128.71, 127.37, 127.20, 126.73, 126.19, 122.90, 121.11, 120.87, 116.13, 115.00, 65.52, 42.37, 30.95, 25.57. MS (EI): m/z (%) = 349 (M^+^, 100.00), 349 (100)；HRMS calculated for [C_25_H_19_NO]^+^: 349.1467 found: 349.1464. Enantiomeric excess was determined by HPLC with a Chiralpak ODH column (hexanes: 2-propanol = 80:20, 0.8 mL/min, 254 nm); minor enantiomer tr = 35.5 min, major enantiomer tr = 30.5 min. [α]_D_^20^ = -83.4 (*c* = 0.4, CHCl_3_).

1. Synthesis of (*S*)-6-methoxy-10b-methyl-1,10b-dihydro-2*H*-indeno[1,2,3-*de*] chromene (**2o)**.

Prepared according to typical procedure **B** from allyl ether **1o** (114.0 mg, 0.3 mmol), after a flash column chromatography (hexanes: EA = 100:1) afforded the product **2o** as a colorless liquid (71.1 mg, 94% yield) with 96:4 er. ^1^H NMR (400 MHz, CDCl_3_) δ 8.01 (d, *J* = 7.6 Hz, 1 H), 7.41 (d, *J* = 7.4 Hz, 1 H), 7.40 – 7.29 (m, 1 H), 7.27 – 7.23 (m, 1 H), 6.78 (d, *J* = 8.7 Hz, 1 H), 6.69 (d, *J* = 8.7 Hz, 1 H), 4.65 – 4.58 (m, 1 H), 4.54 – 4.49 (m, 1 H), 3.94 (s, 3 H), 2.33 – 2.28 (m, 1 H), 1.66 (td, *J* = 13.1, 6.5 Hz, 1 H), 1.46 (s, 3 H). ^13^C NMR (400 MHz, CDCl_3_) δ 153.02, 149.91, 146.55, 139.32, 136.36, 127.36, 126.10, 124.00, 122.43, 114.00, 111.78, 65.30, 56.03, 43.00, 30.98, 25.19. MS (EI): m/z (%) = 252 (M^+^, 73.72), 237 (100)；HRMS calculated for [C_17_H_16_O_2_]^+^: 252.1150 found: 252.1157. Enantiomeric excess was determined by HPLC with a Chiralpak ODH column (hexanes: 2-propanol = 99:1, 0.5 mL/min, 254 nm); minor enantiomer tr = 14.6 min, major enantiomer tr = 16.6 min. [α]_D_^20^ = -203.5 (*c* = 0.4, CHCl_3_).

1. Synthesis of (*S*)-8,10b-dimethyl-1,10b-dihydro-2*H*-indeno[1,2,3-*de*]chromene (**2p)**.

Prepared according to typical procedure **B** from allyl ether **1p** (109.0 mg, 0.3 mmol), after a flash column chromatography (hexanes: Et_2_O = 100:1) afforded the product **2p** as a colorless liquid (63.3 mg, 96% yield) with 93:7 er. ^1^H NMR (400 MHz, CDCl_3_) δ 7.53 (s, 1 H), 7.33 (d, *J* = 7.6 Hz, 1 H), 7.24 – 7.22 (m, 2 H), 7.10 (d, *J* = 7.7 Hz, 1 H), 6.71 (dd, *J* = 6.1, 2.7 Hz, 1 H), 4.68 – 4.61 (m, 1 H), 4.58 – 4.53 (m, 1 H), 2.44 (s, 3 H), 2.32 – 2.28 (m, 1 H), 1.65 (td, *J* = 13.1, 6.3 Hz, 1 H), 1.45 (s, 3 H). ^13^C NMR (400 MHz, CDCl_3_) δ 152.74, 150.89, 140.43, 140.27, 136.99, 135.00, 129.20, 127.66, 122.64, 121.66, 113.42, 112.19, 65.49, 42.02, 30.99, 25.45, 21.48. MS (EI): m/z (%) = 236 (M^+^, 47.50), 221 (100)；HRMS calculated for [C_17_H_16_O]^+^: 236.1201 found: 236.1204. Enantiomeric excess was determined by HPLC with a Chiralpak ODH column (hexanes: 2-propanol = 99:1, 0.5 mL/min, 254 nm); minor enantiomer tr = 10.9 min, major enantiomer tr = 11.7 min. [α]_D_^20^ = -28.7 (*c* = 0.3, CHCl_3_).

1. Synthesis of (*S*)-8-methoxy-10b-methyl-1,10b-dihydro-2*H*-indeno[1,2,3-*de*] chromene (**2q)**.

Prepared according to typical procedure **A** from allyl ether **1q** (76.0 mg, 0.2 mmol), after a flash column chromatography (hexanes: Et_2_O = 100:1) afforded the product **2q** as a colorless liquid (43.8 mg, 87% yield) with 92.5:7.5 er. ^1^H NMR (400 MHz, CDCl_3_) δ 7.33 (d, *J* = 8.2 Hz, 1 H), 7.26 – 7.20 (m, 3 H), 6.83 (dd, *J* = 8.2, 2.4 Hz, 1 H), 6.72 (dd, *J* = 7.1, 1.6 Hz, 1 H), 4.64 (td, *J* = 12.4, 4.0 Hz, 1 H), 4.55 (dd, *J* = 11.4, 6.3 Hz, 1 H), 3.88 (s, 3 H), 2.29 (dd, *J* = 13.0, 4.0 Hz, 1 H), 1.64 (td, *J* = 13.0, 6.2 Hz, 1 H), 1.45 (s, 3 H). ^13^C NMR (400 MHz, CDCl_3_) δ 159.51, 152.77, 146.04, 141.56, 140.27, 135.55, 129.21, 123.49, 113.70, 112.83, 112.24, 106.40, 65.64, 55.57, 41.78, 31.17, 25.47. MS (EI): m/z (%) = 252 (M^+^, 36.25), 237 (100)；HRMS calculated for [C_17_H_16_O_2_]^+^: 252.1150 found: 252.1149. Enantiomeric excess was determined by HPLC with a Chiralpak ODH column (hexanes: 2-propanol = 99:1, 0.5 mL/min, 220 nm); minor enantiomer tr = 26.5 min, major enantiomer tr = 19.5 min. [α]_D_^20^ = -33.9 (*c* = 0.4, CHCl_3_).

1. Synthesis of (*S*)-10b-methyl-8-phenyl-1,10b-dihydro-2*H*-indeno[1,2,3-*de*]

chromene (**2r)**.

Prepared according to typical procedure **B** from allyl ether **1r** (85.2 mg, 0.2 mmol), after a flash column chromatography (hexanes: Et_2_O = 100:1) afforded the product **2r** as a colorless liquid (46.3 mg, 78% yield) with 92:8 er. ^1^H NMR (400 MHz, CDCl_3_) δ 7.93 (s, 1 H), 7.68 (dd, *J* = 7.8, 1.7 Hz, 2 H), 7.54 – 7.49 (m, 4 H), 7.41 – 7.37 (m, 1 H), 7.33 – 7.26 (m, 2 H), 6.77 (d, *J* = 7.6 Hz, 1H), 4.72 – 4.57 (m, 1 H), 4.60 (dd, *J* = 11.4, 6.1 Hz, 1 H), 2.36 (dd, *J* = 13.0, 3.9 Hz, 1 H), 1.72 (td, *J* = 13.0, 6.2 Hz, 1 H), 1.52 (s, 3 H). ^13^C NMR (400 MHz, CDCl_3_) δ 152.79, 152.70, 141.40, 140.81, 140.76, 140.23, 135.00, 129.36, 128.73, 127.26, 127.21, 126.07, 123.18, 119.81, 113.72, 112.36, 65.45, 42.24, 30.90, 25.48. MS (EI): m/z (%) = 298 (M^+^, 47.88), 283 (100)；HRMS calculated for [C_22_H_18_O]^+^: 298.1358 found: 298.1351. Enantiomeric excess was determined by HPLC with a Chiralpak ODH column (hexanes: 2-propanol = 99:1, 0.5 mL/min, 254 nm); minor enantiomer tr = 17.9 min, major enantiomer tr = 25.5 min. [α]_D_^20^ = 18.0 (*c* = 0.4, CHCl_3_).

1. Synthesis of (*S*)-5-(tert-butyl)-8,10b-dimethyl-1,10b-dihydro-2*H*-indeno [1,2,3-*de*]chromene (**2s)**.

Prepared according to typical procedure **B** from allyl ether **1s** (84.0 mg, 0.2 mmol), after a flash column chromatography (hexanes: Et_2_O = 100:1) afforded the product **2s** as a colorless liquid (54.2 mg, 92% yield) with 95.5:4.5 er. ^1^H NMR (400 MHz, CDCl_3_) δ 7.54 (dt, *J* = 1.6, 0.7 Hz, 1 H), 7.29 (d, *J* = 7.7 Hz, 1 H), 7.27 (d, *J* = 1.3 Hz, 1 H), 7.07 (ddd, *J* = 7.7, 1.7, 0.8 Hz, 1 H), 6.75 (d, *J* = 1.3 Hz, 1 H), 4.63 (ddd, *J* = 13.2, 11.4, 3.9 Hz, 1 H), 4.53 (ddd, *J* = 11.4, 6.1, 1.2 Hz, 1 H), 2.43 (s, 3 H), 2.25 (ddd, *J* = 13.0, 3.9, 1.2 Hz, 1 H), 1.62 (td, *J* = 13.1, 6.1 Hz, 1 H), 1.44 (s, 3 H), 1.36 (s, 9 H). ^13^C NMR (400 MHz, CDCl_3_) δ 153.43, 152.35, 151.38, 140.58, 140.20, 136.89, 132.24, 127.44, 122.66, 121.48, 110.65, 109.32, 65.54, 41.76, 35.06, 31.72, 31.25, 25.65, 21.51. MS (EI): m/z (%) = 292 (M^+^, 35.05), 277 (100)；HRMS calculated for [C_21_H_24_O]^+^: 292.1827 found: 292.1828. Enantiomeric excess was determined by HPLC with a Chiralpak ODH column (hexanes: 2-propanol = 99.5:0.5, 0.5 mL/min, 254 nm); minor enantiomer tr = 8.4 min, major enantiomer tr = 9.3 min. [α]_D_^20^ = -45.3 (*c* = 0.3, CHCl_3_).

1. Synthesis of (*S*)-5-(tert-butyl)-8-methoxy-10b-methyl-1,10b-dihydro-2*H*-indeno

[1,2,3-*de*]chromene (**2t)**.

Prepared according to typical procedure **B** from allyl ether **1t** (87.2 mg, 0.2 mmol), after a flash column chromatography (hexanes: Et_2_O = 100:1) afforded the product **2t** as a colorless liquid (54.7 mg, 89% yield) with 93:7 er. ^1^H NMR (300 MHz, CDCl_3_) δ 7.30 (d, *J* = 8.3 Hz, 1 H), 7.25 (dd, *J* = 3.1, 1.5 Hz, 2 H), 6.81 (dd, *J* = 8.2, 2.5 Hz, 1 H), 6.77 (d, *J* = 1.3 Hz, 1 H), 4.62 (ddd, *J* = 13.0, 11.4, 3.8 Hz, 1 H), 4.53 (ddd, *J* = 11.4, 6.2, 1.2 Hz, 1 H), 3.88 (s, 3 H), 2.25 (ddd, *J* = 12.9, 3.8, 1.2 Hz, 1 H), 1.63 (dt, *J* = 13.4, 6.7 Hz, 1 H), 1.44 (s, 3 H), 1.36 (s, 9 H). ^13^C NMR (400 MHz, CDCl_3_) δ 159.46, 153.43, 152.36, 146.49, 141.83, 139.99, 132.76, 123.47, 112.73, 110.93, 109.30, 106.08, 65.63, 55.58, 41.47, 35.07, 31.71, 31.39, 25.62. MS (EI): m/z (%) = 308 (M^+^, 27.74), 293 (100)；HRMS calculated for [C_21_H_24_O_2_]^+^: 308.1776 found: 308.1781. Enantiomeric excess was determined by HPLC with a Chiralpak ODH column (hexanes: 2-propanol = 99:1, 0.5 mL/min, 254 nm); minor enantiomer tr = 9.3 min, major enantiomer tr = 10.0 min. [α]_D_^20^ = -42.4 (*c* = 0.4, CHCl_3_).

1. Synthesis of (*S*)-5-(tert-butyl)-10b-methyl-8-phenyl-1,10b-dihydro-2*H*-indeno

[1,2,3-*de*]chromene (**2u)**.

Prepared according to typical procedure **B** from allyl ether **1u** (96.4 mg, 0.2 mmol), after a flash column chromatography (hexanes: Et_2_O = 100:1) afforded the product **2u** as a colorless liquid (62.3 mg, 88% yield) with 96:4 er. ^1^H NMR (400 MHz, CDCl_3_) δ 7.94 (s, 1 H), 7.70 – 7.68 (m, 2 H), 7.52 – 7.46 (m, 4 H), 7.40 – 7.36 (m, 2 H), 6.81 (d, *J* = 1.6 Hz, 1 H), 4.71 – 4.64 (m, 1 H), 4.60 – 4.56 (m, 1 H), 2.35 – 2.29 (m, 1 H), 1.71 (td, *J* = 13.1, 6.0 Hz, 1 H), 1.52 (s, 3 H), 1.39 (s, 9 H). ^13^C NMR (400 MHz, CDCl_3_) δ 153.63, 153.20, 152.40, 141.51, 141.11, 140.66, 139.99, 132.24, 128.72, 127.18, 125.85, 123.20, 119.65, 65.48, 41.96, 35.10, 31.72, 31.14, 25.66. MS (EI): m/z (%) = 354 (M^+^, 41.03), 339 (100)；HRMS calculated for [C_26_H_26_O]^+^: 354.1984 found: 354.1989. Enantiomeric excess was determined by HPLC with a Chiralpak ODH column (hexanes: 2-propanol = 99:1, 0.5 mL/min, 254 nm); minor enantiomer tr = 10.3 min, major enantiomer tr = 15.5 min. [α]_D_^20^ = 11.8 (*c* = 0.4, CHCl_3_).

1. Synthesis of (*S*)-5-(tert-butyl)-8-fluoro-10b-methyl-1,10b-dihydro-2*H*-indeno

[1,2,3-*de*]chromene (**2v)**.

Prepared according to typical procedure **B** from allyl ether **1v** (59.4 mg, 0.14 mmol), after a flash column chromatography (hexanes: Et_2_O = 100:1) afforded the product **2v** as a white solid (37.7 mg, 91% yield) with 90:10 er. Mp: 122-123 ^o^C. ^1^H NMR (300 MHz, CDCl_3_) δ 7.40-7.31 (m, 2 H), 7.24 (d, *J* = 1.3 Hz, 1 H), 6.93 (ddd, *J* = 9.2, 8.2, 2.4 Hz, 1 H), 6.79 (d, *J* = 1.3 Hz, 1 H), 4.63 (ddd, *J* = 13.0, 11.5, 3.8 Hz, 1 H), 4.54 (ddd, *J* = 11.5, 6.3, 1.3 Hz, 1 H), 2.27 (ddd, *J* = 13.0, 3.8, 1.3 Hz, 1 H), 1.64 (dt, *J* = 13.1, 6.6 Hz, 1 H), 1.44 (s, 3 H), 1.36 (s, 9 H). ^19^F NMR (400 MHz, CDCl_3_) δ -115.66. ^13^C NMR (400 MHz, CDCl_3_) δ 162.71 (d, *J* = 243.7 Hz), δ 153.71, 152.38, 149.53 (d, *J* = 2.6 Hz), 142.50, 139.18 (d, *J* = 3.1 Hz), 132.71, 123.79 (d, *J* = 9.2 Hz), 113.18 (d, *J* = 22.9 Hz), 111.41, 109.58, 107.99 (d, *J* = 23.0 Hz), 65.52, 41.71, 35.10, 31.68, 31.11, 25.63. MS (EI): m/z (%) = 296 (M^+^, 32.67), 281 (100)；HRMS calculated for [C_20_H_21_OF]^+^: 296.1576 found: 296.1579. Enantiomeric excess was determined by HPLC with a Chiralpak ODH column (hexanes: 2-propanol = 99.5:0.5, 0.5 mL/min, 254 nm); minor enantiomer tr = 9.1 min, major enantiomer tr = 9.7 min. [α]_D_^20^ = -63.1 (*c* = 0.3, CHCl_3_).

1. Synthesis of (*S*)-5-(tert-butyl)-8-chloro-10b-methyl-1,10b-dihydro-2*H*-indeno

[1,2,3-*de*]chromene (**2w)**.

Prepared according to typical procedure **B** from allyl ether **1w** (88.1 mg, 0.2 mmol), after a flash column chromatography (hexanes: Et_2_O = 100:1) afforded the product **2w** as a colorless liquid (57.5 mg, 92% yield) with 92.5:7.5 er. ^1^H NMR (400 MHz, CDCl_3_) δ 7.68 (d, *J* = 1.9 Hz, 1 H), 7.32 (d, *J* = 8.0 Hz, 1 H), 7.25 (d, *J* = 1.3 Hz, 1 H), 7.21 (dd, *J* = 8.0, 2.0 Hz, 1 H), 6.79 (d, *J* = 1.3 Hz, 1 H), 4.62 (ddd, *J* = 13.1, 11.5, 3.9 Hz, 1 H), 4.54 (ddd, *J* = 11.5, 6.1, 1.2 Hz, 1 H), 2.26 (ddd, *J* = 13.0, 3.9, 1.2 Hz, 1 H), 1.63 (td, *J* = 13.0, 6.1 Hz, 1 H), 1.43 (s, 3 H), 1.35 (s, 9 H). ^13^C NMR (400 MHz, CDCl_3_) δ 153.80, 152.40, 152.35, 142.33, 138.90, 133.16, 132.27, 126.46, 123.94, 121.14, 111.47, 109.63, 65.41, 41.94, 35.10, 31.67, 30.93, 25.56. MS (EI): m/z (%) = 312 (M^+^, 33.80), 297 (100)；HRMS calculated for [C_20_H_21_OCl]^+^: 312.1281 found: 312.1285. Enantiomeric excess was determined by HPLC with a Chiralpak ODH column (hexanes: 2-propanol = 99.5:0.5, 0.5 mL/min, 254 nm); minor enantiomer tr = 8.9 min, major enantiomer tr = 9.7 min. [α]_D_^20^ = -26.6 (*c* = 0.4, CHCl_3_).

1. Synthesis of (*S*)-5-(tert-butyl)-10b-methyl-1,10b-dihydro-2*H*-indeno[1,2,3-*de*] chromene-8-carbonitrile (**2x)**.

Prepared according to typical procedure **B** from allyl ether **1x** (75.0 mg, 0.2 mmol), after a flash column chromatography (hexanes: EA = 20:1) afforded the product **2x** as a colorless liquid (25.8 mg, 52% yield) with 90.5:9.5 er. ^1^H NMR (400 MHz, CDCl_3_) δ 7.97 (dd, *J* = 1.5, 0.7 Hz, 1 H), 7.55 (dd, *J* = 7.7, 1.5 Hz, 1 H), 7.50 (dd, *J* = 7.7, 0.7 Hz, 1 H), 7.30 (d, *J* = 1.3 Hz, 1 H), 6.84 (d, *J* = 1.3 Hz, 1 H), 4.64 (ddd, *J* = 13.0, 11.5, 3.8 Hz, 1 H), 4.57 (ddd, *J* = 11.6, 6.2, 1.2 Hz, 1 H), 2.31 (ddd, *J* = 12.9, 3.8, 1.2 Hz, 1 H), 1.67 (td, *J* = 13.0, 6.2 Hz, 1 H), 1.46 (s, 3 H), 1.36 (s, 9 H). ^13^C NMR (400 MHz, CDCl_3_) δ 158.72, 154.23, 152.44, 141.64, 137.94, 131.73, 130.57, 124.31, 123.75, 119.23, 112.10, 111.17, 109.88, 65.15, 42.79, 35.15, 31.62, 30.44, 25.48. MS (EI): m/z (%) = 303 (M^+^, 33.53), 288 (100)；HRMS calculated for [C_21_H_21_NO]^+^: 303.1623 found: 303.1624. Enantiomeric excess was determined by HPLC with a Chiralpak ODH column (hexanes: 2-propanol = 99:1, 0.5 mL/min, 254 nm); minor enantiomer tr = 15.4 min, major enantiomer tr = 16.4 min. [α]_D_^20^ = -28.6 (*c* = 0.4, CHCl_3_).

1. Synthesis of methyl (*S*)-5-(tert-butyl)-10b-methyl-1,10b-dihydro-2*H*-indeno [1,2,3-*de*]chromene-8-carboxylate (**2y)**.

Prepared according to typical procedure **B** from allyl ether **1y** (92.8 mg, 0.2 mmol), after a flash column chromatography (hexanes: EA = 40:1) afforded the product **2y** as a colorless liquid (52.4 mg, 78% yield) with 94:6 er. ^1^H NMR (300 MHz, CDCl_3_) δ 8.37 (d, *J* = 1.5 Hz, 1 H), 7.97 (dd, *J* = 7.9, 1.6 Hz, 1 H), 7.47 (d, *J* = 7.9 Hz, 1 H), 7.36 (d, *J* = 1.3 Hz, 1 H), 6.80 (d, *J* = 1.3 Hz, 1 H), 4.65 (ddd, *J* = 12.9, 11.5, 3.8 Hz, 1 H), 4.55 (ddd, *J* = 11.5, 6.2, 1.2 Hz, 1 H), 3.96 (s, 3 H), 2.30 (ddd, *J* = 13.0, 3.8, 1.2 Hz, 1 H), 1.67 (dq, *J* = 12.9, 6.2 Hz, 1 H), 1.46 (s, 3 H), 1.37 (s, 9 H). ^13^C NMR (400 MHz, CDCl_3_) δ 167.23, 158.98, 153.90, 152.36, 140.92, 139.13, 131.84, 129.40, 128.34, 122.83, 121.96, 111.32, 109.80, 65.24, 52.12, 42.42, 35.12, 31.67, 30.68, 25.58. MS (EI): m/z (%) = 336 (M^+^, 41.84), 321 (100)；HRMS calculated for [C_22_H_24_O_3_]^+^: 336.1725 found: 336.1722. Enantiomeric excess was determined by HPLC with a Chiralpak ODH column (hexanes: 2-propanol = 98:2, 0.5 mL/min, 254 nm); minor enantiomer tr = 12.1 min, major enantiomer tr = 14.4 min. [α]_D_^20^ = -17.6 (*c* = 0.4, CHCl_3_).

1. Synthesis of (*S*)-5-(tert-butyl)-9,10b-dimethyl-1,10b-dihydro-2*H*-indeno [1,2,3-*de*]chromene (**2z)**.

Prepared according to typical procedure **B** from allyl ether **1z** (84.0 mg, 0.2 mmol), after a flash column chromatography (hexanes: Et_2_O = 100:1) afforded the product **2z** as a colorless liquid (53.9 mg, 92% yield) with 96:4 er. ^1^H NMR (300 MHz, CDCl_3_) δ 7.59 (d, *J* = 7.7 Hz, 1 H), 7.24 (d, *J* = 1.2 Hz, 1 H), 7.22 (dd, *J* = 2.0, 1.1 Hz, 1 H), 7.13 (ddd, *J* = 7.8, 1.7, 0.8 Hz, 1 H), 6.74 (t, *J* = 1.2 Hz, 1 H), 4.62 (ddd, *J* = 13.0, 11.4, 3.8 Hz, 1 H), 4.52 (ddd, *J* = 11.5, 6.2, 1.2 Hz, 1 H), 2.41 (s, 3 H), 2.24 (ddd, *J* = 13.0, 3.8, 1.1 Hz, 1 H), 1.63 (td, *J* = 13.0, 6.2 Hz, 1 H), 1.44 (s, 3 H), 1.35 (s, 9 H). ^13^C NMR (400 MHz, CDCl_3_) δ 154.38, 153.42, 152.32, 140.23, 137.77, 136.51, 131.71, 127.95, 123.80, 120.52, 110.32, 109.10, 65.45, 41.93, 35.05, 31.72, 31.15, 25.68, 21.64. MS (EI): m/z (%) = 292 (M^+^, 44.03), 277 (100)；HRMS calculated for [C_21_H_24_O]^+^: 292.1827 found: 292.1830. Enantiomeric excess was determined by HPLC with a Chiralpak ODH column (hexanes: 2-propanol = 99:1, 0.5 mL/min, 254 nm); minor enantiomer tr = 7.6 min, major enantiomer tr = 7.9 min. [α]_D_^20^ = -98.4 (*c* = 0.4, CHCl_3_).

1. Synthesis of (*S*)-5-(tert-butyl)-9-methoxy-10b-methyl-1,10b-dihydro-2*H*-indeno

[1,2,3-*de*]chromene (**2aa)**.

Prepared according to typical procedure **B** from allyl ether **1aa** (87.2 mg, 0.2 mmol), after a flash column chromatography (hexanes: EA = 50:1) afforded the product **2aa** as a white solid (44.6 mg, 72% yield) with 95:5 er. Mp: 138-140 ^o^C. ^1^H NMR (300 MHz, CDCl_3_) δ 7.61 (d, *J* = 8.3 Hz, 1 H), 7.20 (d, *J* = 1.4 Hz, 1 H), 6.96 (d, *J* = 2.4 Hz, 1 H), 6.87 (dd, *J* = 8.3, 2.4 Hz, 1 H), 6.71 (d, *J* = 1.4 Hz, 1 H), 4.62 (ddd, *J* = 13.0, 11.4, 3.8 Hz, 1 H), 4.52 (ddd, *J* = 11.5, 6.2, 1.2 Hz, 1 H), 3.85 (s, 3 H), 2.23 (ddd, *J* = 13.0, 3.8, 1.3 Hz, 1 H), 1.66 (td, *J* = 13.1, 6.3 Hz, 1 H), 1.44 (s, 3 H), 1.35 (s, 9 H). ^13^C NMR (400 MHz, CDCl_3_) δ 159.11, 156.04, 153.50, 152.22, 140.08, 133.30, 131.37, 121.44, 112.33, 109.75, 109.44, 108.66, 65.35, 55.50, 42.11, 35.04, 31.71, 31.10, 25.79. MS (EI): m/z (%) = 308 (M^+^, 77.85), 293 (100)；HRMS calculated for [C_21_H_24_O_2_]^+^: 308.1776 found: 308.1779. Enantiomeric excess was determined by HPLC with a Chiralpak IC column (hexanes: 2-propanol = 99:1, 0.5 mL/min, 254 nm); minor enantiomer tr = 8.6 min, major enantiomer tr = 8.2 min. [α]_D_^20^ = -90.5 (*c* = 0.4, CHCl_3_).

1. Synthesis of (*S*)-5-(tert-butyl)-10b-methyl-9-phenyl-1,10b-dihydro-2*H*-indeno [1,2,3-*de*]chromene (**2ab)**.

Prepared according to typical procedure **C** from allyl ether **1ab** (96.5 mg, 0.2 mmol), after a flash column chromatography (hexanes: Et_2_O = 200:1) afforded the product **2ab** as a white solid (62.4 mg, 88% yield) with 95:5 er. Mp: 150-151 ^o^C. ^1^H NMR (300 MHz, CDCl_3_) δ 7.77 (dd, *J* = 7.9, 0.7 Hz, 1 H), 7.66 (d, *J* = 1.5 Hz, 1 H), 7.63 (q, *J* = 1.2, 0.8 Hz, 2 H), 7.58 (dd, *J* = 7.9, 1.7 Hz, 1 H), 7.48-7.42 (m, 2 H), 7.37-7.34 (m, 1 H), 7.31 (d, *J* = 1.4 Hz, 1 H), 6.78 (d, *J* = 1.3 Hz, 1 H), 4.66 (ddd, *J* = 13.0, 11.4, 3.8 Hz, 1 H), 4.56 (ddd, *J* = 11.4, 6.2, 1.2 Hz, 1 H), 2.32 (ddd, *J* = 13.0, 3.8, 1.2 Hz, 1 H), 1.71 (td, *J* = 13.0, 6.1 Hz, 1 H), 1.50 (s, 3 H), 1.37 (s, 9 H). ^13^C NMR (400 MHz, CDCl_3_) δ 154.85, 153.64, 152.37, 141.44, 139.82, 139.68, 132.13, 128.76, 127.15, 126.39, 121.82, 121.05, 110.83, 109.51, 65.47, 42.23, 35.10, 31.72, 31.13, 25.76. MS (EI): m/z (%) = 354 (M^+^, 6.47), 291 (100)；HRMS calculated for [C_26_H_26_O]^+^: 354.1984 found: 354.1977. Enantiomeric excess was determined by HPLC with a Chiralpak ODH column (hexanes: 2-propanol = 99:1, 0.5 mL/min, 254 nm); minor enantiomer tr = 10.5 min, major enantiomer tr = 11.9 min. [α]_D_^20^ = -100.8 (*c* = 0.4, CHCl_3_).

1. Synthesis of (*S*)-5-(tert-butyl)-8,9,10b-trimethyl-1,10b-dihydro-2*H*-indeno [1,2,3-*de*]chromene (**2ac)**.

Prepared according to typical procedure **B** from allyl ether **1ac** (86.9 mg, 0.2 mmol), after a flash column chromatography (hexanes: Et_2_O = 200:1) afforded the product **2ac** as a white solid (38.6 mg, 64% yield) with 95.5:4.5 er. Mp: 135-137 ^o^C. ^1^H NMR (300 MHz, CDCl_3_) δ 7.50 (s, 1 H), 7.24 (d, *J* = 1.4 Hz, 1 H), 7.18 (s, 1 H), 6.73 (d, *J* = 1.3 Hz, 1 H), 4.62 (ddd, *J* = 13.0, 11.4, 3.8 Hz, 1 H), 4.52 (ddd, *J* = 11.5, 6.2, 1.2 Hz, 1 H), 2.33 (s, 3 H), 2.32 (s, 3 H), 2.24 (ddd, *J* = 12.9, 3.8, 1.2 Hz, 1 H), 1.62 (dt, *J* = 13.1, 6.6 Hz, 1 H), 1.43 (s, 3 H), 1.35 (s, 9 H). ^13^C NMR (400 MHz, CDCl_3_) δ 153.35, 152.31, 151.93, 140.41, 138.23, 135.38, 135.07, 131.88, 124.23, 121.94, 110.23, 109.07, 65.53, 41.78, 35.05, 31.73, 31.33, 25.66, 20.19, 20.04. MS (EI): m/z (%) = 306 (M^+^, 41.18), 291 (100)；HRMS calculated for [C_22_H_26_O]^+^: 306.1984 found: 306.1992. Enantiomeric excess was determined by HPLC with a Chiralpak ODH column (hexanes: 2-propanol = 99:1, 0.5 mL/min, 254 nm); minor enantiomer tr = 7.8 min, major enantiomer tr = 8.6 min. [α]_D_^20^ = -65.3 (*c* = 0.4, CHCl_3_).

1. Synthesis of (*S*)-5-(tert-butyl)-12b-methyl-1,12b-dihydro-2*H*-benzo[5,6]indeno

[1,2,3-*de*]chromene (**2ad)**.

Prepared according to typical procedure **C** from allyl ether **1ad** (91.2 mg, 0.2 mmol), after a flash column chromatography (hexanes: Et_2_O = 100:1) afforded the product **2ad** as a white solid (59.6 mg, 91% yield) with 92.5:7.5 er. Mp: 197-198 ^o^C. ^1^H NMR (300 MHz, CDCl_3_) δ 8.12 (s, 1 H), 7.91-7.88 (m, 1 H), 7.84-7.81 (m, 1 H), 7.79 (s, 1 H), 7.46-7.42 (m, 3 H), 6.82 (d, *J* = 1.4 Hz, 1 H), 4.67 (ddd, *J* = 13.0, 11.4, 3.8 Hz, 1 H), 4.57 (ddd, *J* = 11.4, 6.2, 1.3 Hz, 1 H), 2.38 (ddd, *J* = 13.0, 3.8, 1.3 Hz, 1 H), 1.80 (td, *J* = 13.0, 6.2 Hz, 1 H), 1.52 (s, 3 H), 1.39 (s, 9 H). ^13^C NMR (400 MHz, CDCl_3_) δ 153.70, 152.39, 152.22, 139.41, 139.28, 133.42, 132.87, 132.46, 128.21, 127.90, 125.60, 125.55, 121.35, 119.01, 111.41, 109.89, 65.38, 41.58, 35.14, 31.72, 31.59, 26.83. MS (EI): m/z (%) = 328 (M^+^, 36.18), 313 (100)；HRMS calculated for [C_24_H_24_O]^+^: 328.1827 found: 328.1833. Enantiomeric excess was determined by HPLC with a Chiralpak ODH column (hexanes: 2-propanol = 99:1, 0.5 mL/min, 254 nm); minor enantiomer tr = 9.4 min, major enantiomer tr = 10.2 min. [α]_D_^20^ = -2.8 (*c* = 0.4, CHCl_3_).

1. Synthesis of (*S*)-5-(tert-butyl)-13b-methyl-1,13b-dihydro-2*H*-spiro[benzo[5,6]-s-

indaceno[1,2,3-*de*]chromene-12,9'-fluorene] (**2ae)**.

Prepared according to typical procedure **C** from allyl ether **1ae** (128.9 mg, 0.2 mmol), after a flash column chromatography (hexanes: Et_2_O = 100:1) afforded the product **2ae** as a white solid (63.4 mg, 62% yield) with 96:4 er. Mp: 128-129 ^o^C. ^1^H NMR (300 MHz, CDCl_3_) δ 8.17 (d, *J* = 0.8 Hz, 1 H), 7.91 (dt, *J* = 7.6, 0.9 Hz, 1 H), 7.85 (ddt, *J* = 7.6, 6.7, 1.0 Hz, 2 H), 7.40 (dd, *J* = 8.7, 1.2 Hz, 2 H), 7.37-7.31 (m, 2 H), 7.14 (td, *J* = 7.5, 1.2 Hz, 1 H), 7.09 (dd, *J* = 7.4, 1.1 Hz, 1 H), 7.04 (dd, *J* = 7.4, 1.1 Hz, 1 H), 6.85 (dt, *J* = 7.7, 1.0 Hz, 1 H), 6.76 (d, *J* = 1.0 Hz, 2 H), 6.67 (ddt, *J* = 7.5, 6.5, 0.9 Hz, 2 H), 4.56-4.40 (m, 2 H), 2.04-1.98 (m, 1 H), 1.52-1.47 (m, 1 H), 1.40 (s, 9 H), 1.29 (s, 3 H). ^13^C NMR (400 MHz, CDCl_3_) δ 154.79, 153.59, 152.28, 149.22, 149.10, 148.96, 147.74, 141.85, 141.63, 141.60, 141.39, 140.54, 139.95, 132.21, 127.92, 127.81, 127.71, 127.67, 127.59, 127.54, 124.28, 124.09, 123.82, 119.88, 119.78, 118.73, 112.08, 110.74, 109.32, 65.89, 65.39, 41.97, 35.12, 31.76, 31.09, 25.85. MS (EI): m/z (%) = 516 (M^+^, 2.64), 291 (100)；HRMS calculated for [C_39_H_32_O]^+^: 516.2453 found: 516.2461. Enantiomeric excess was determined by HPLC with a Chiralpak IC column (hexanes: 2-propanol = 99:1, 0.5 mL/min, 254 nm); minor enantiomer tr = 8.0 min, major enantiomer tr = 8.4 min. [α]_D_^20^ = -71.3 (*c* = 0.4, CHCl_3_).

1. Synthesis of (*S*)-5-(tert-butyl)-13b-methyl-1,13b-dihydro-2*H*-benzofuro[2',3':5,6] indeno[1,2,3-*de*]chromene (**2af**).

Prepared according to typical procedure **C** from allyl ether **1af** (99.3 mg, 0.2 mmol), after a flash column chromatography (hexanes: Et_2_O = 200:1) afforded the product **2af** as a white solid (61 mg, 83% yield) with 90:10 er. Mp: 212-214 ^o^C. ^1^H NMR (300 MHz, CDCl_3_) δ 7.93 (dd, *J* = 7.2, 0.8 Hz, 2 H), 7.86 (d, *J* = 0.7 Hz, 1 H), 7.57 (dt, *J* = 8.2, 0.9 Hz, 1 H), 7.43 (ddd, *J* = 8.2, 7.3, 1.4 Hz, 1 H), 7.36-7.30 (m, 2 H), 6.81 (d, *J* = 1.3 Hz, 1 H), 4.68 (ddd, *J* = 13.0, 11.4, 3.8 Hz, 1 H), 4.58 (ddd, *J* = 11.4, 6.2, 1.2 Hz, 1 H), 2.38 (ddd, *J* = 13.0, 3.9, 1.2 Hz, 1 H), 1.74 (td, *J* = 13.0, 6.2 Hz, 1 H), 1.53 (s, 3 H), 1.39 (s, 9 H). ^13^C NMR (400 MHz, CDCl_3_) δ 156.85, 156.28, 153.71, 152.34, 149.39, 140.55, 139.77, 132.68, 126.69, 124.47, 122.97, 122.65, 120.19, 114.65, 111.64, 111.15, 109.46, 104.14, 65.60, 41.62, 35.13, 31.72, 31.54, 26.27. MS (EI): m/z (%) = 368 (M^+^, 60.34), 353 (100)；HRMS calculated for [C_26_H_24_O_2_]^+^: 368.1776 found: 368.1775. Enantiomeric excess was determined by HPLC with a Chiralpak ODH column (hexanes: 2-propanol = 99:1, 0.5 mL/min, 254 nm); minor enantiomer tr = 11.0 min, major enantiomer tr = 12.3 min. [α]_D_^20^ = -69.9 (*c* = 0.4, CHCl_3_).

1. Synthesis of (*S*)-5-(tert-butyl)-13b-methyl-8-phenyl-1,2,8,13b-tetrahydropyrano

[4',3',2':3,4]indeno[1,2-b]carbazole (**2ag)**.

Prepared according to typical procedure **C** from allyl ether **1ag** (114.3 mg, 0.2 mmol), after a flash column chromatography (hexanes: Et_2_O = 200:1) afforded the product **2ag** as a white solid (63.6 mg, 72% yield) with 91.5:8.5 er. Mp: 226-228 ^o^C. ^1^H NMR (300 MHz, CDCl_3_) δ 8.14-8.11 (m, 2 H), 7.69-7.60 (m, 5 H), 7.52 (ddt, *J* = 8.6, 6.2, 1.7 Hz, 1 H), 7.38-7.36 (m, 2 H), 7.30-7.27 (m, 1 H), 7.26 (d, *J* = 1.3 Hz, 1 H), 6.77 (d, *J* = 1.3 Hz, 1 H), 4.70 (ddd, *J* = 13.0, 11.4, 3.8 Hz, 1 H), 4.62-4.56 (m, 1 H), 2.49 – 2.37 (m, 1 H), 1.77 (td, *J* = 13.0, 6.2 Hz, 1 H), 1.56 (s, 3 H), 1.35 (s, 9 H). ^13^C NMR (400 MHz, CDCl_3_) δ 153.46, 152.31, 146.89, 141.59, 141.00, 140.34, 139.34, 137.84, 132.73, 130.01, 127.57, 127.45, 125.58, 123.37, 122.45, 119.90, 119.87, 114.41, 110.75, 109.73, 109.31, 102.01, 65.72, 41.44, 35.11, 31.89, 31.74, 26.47. MS (EI): m/z (%) = 443 (M^+^, 5.73), 291 (100)；HRMS calculated for [C_32_H_29_NO]^+^: 443.2249 found: 443.2244. Enantiomeric excess was determined by HPLC with a Chiralpak IB column (hexanes: 2-propanol = 99:1, 0.5 mL/min, 254 nm); minor enantiomer tr = 11.3 min, major enantiomer tr = 10.6 min. [α]_D_^20^ = 9.7 (*c* = 0.4, CHCl_3_).

1. **Additional Information Regarding the Photophysical of 2a, 2b, 2g, 2n, 2o, 2u, 2af**
   1. Absorption (solid trace) and emission (dotted trace) spectra of **2a**


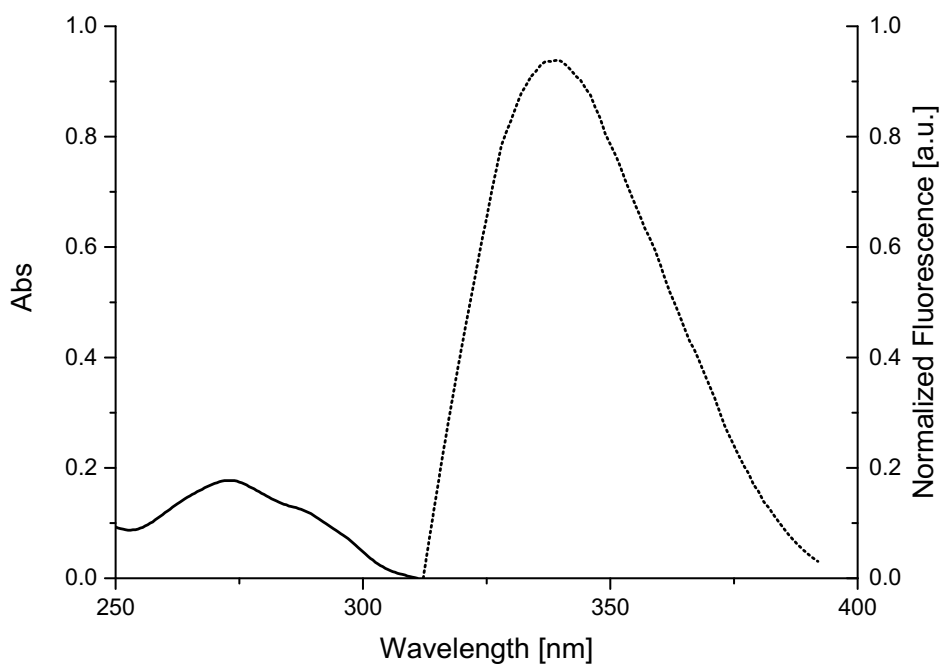


- 1. Absorption (solid trace) and emission (dotted trace) spectra of **2b**


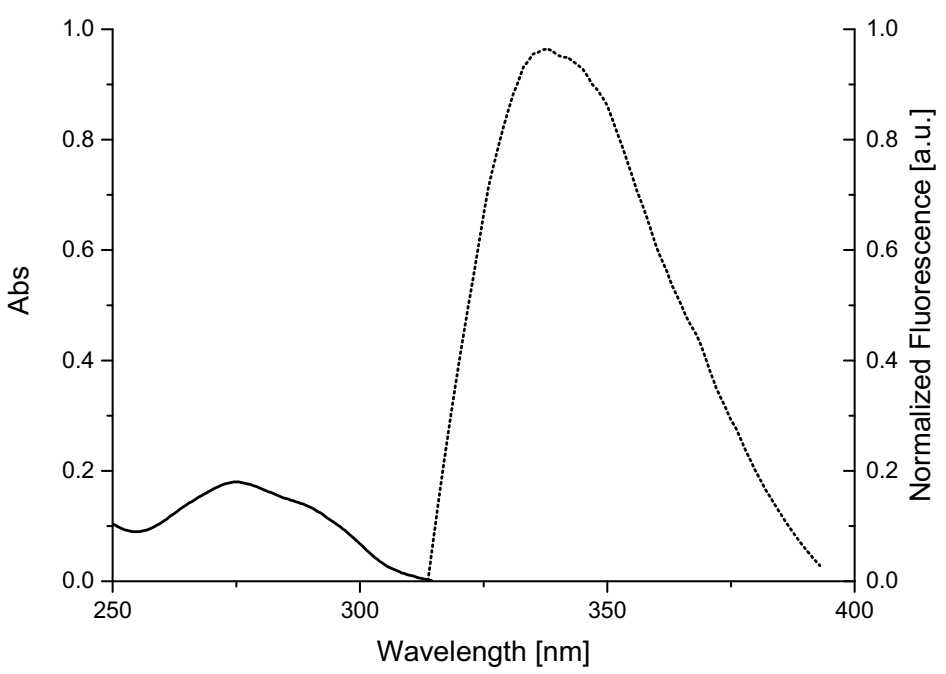


- 1. Absorption (solid trace) and emission (dotted trace) spectra of **2g**


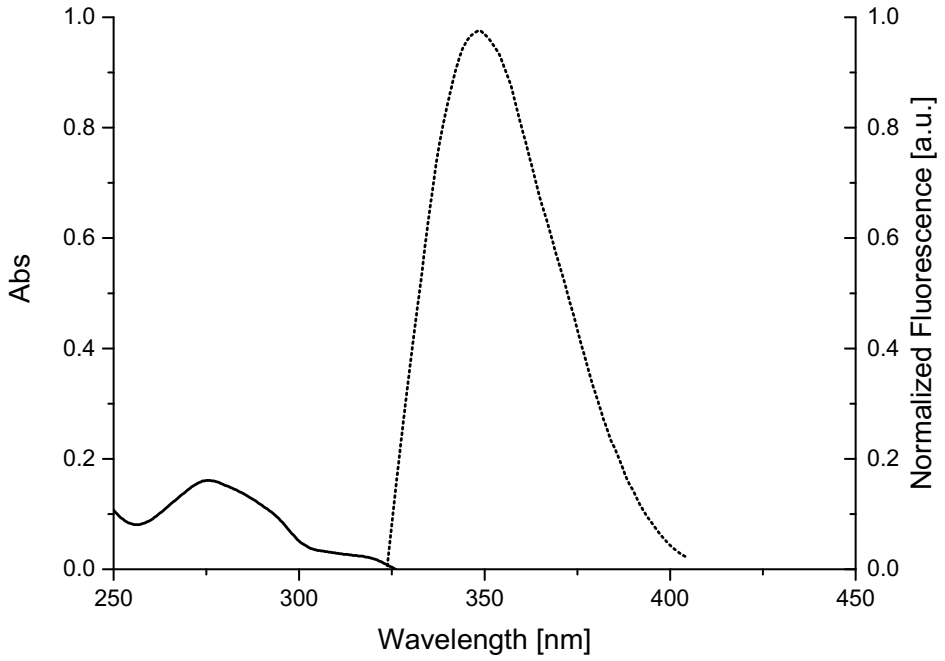


- 1. Absorption (solid trace) and emission (dotted trace) spectra of **2n**


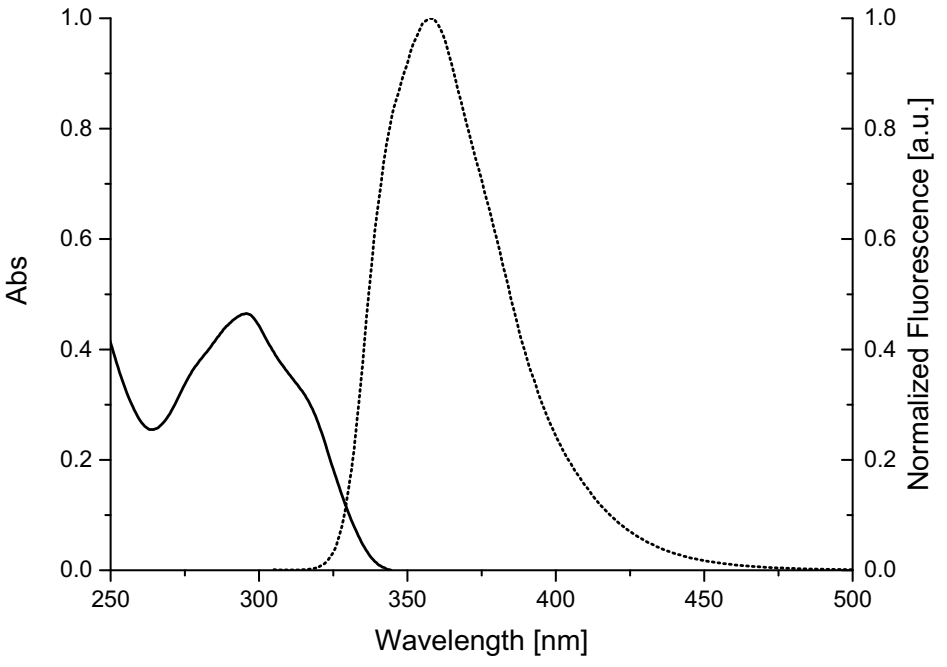


- 1. Absorption (solid trace) and emission (dotted trace) spectra of **2o**


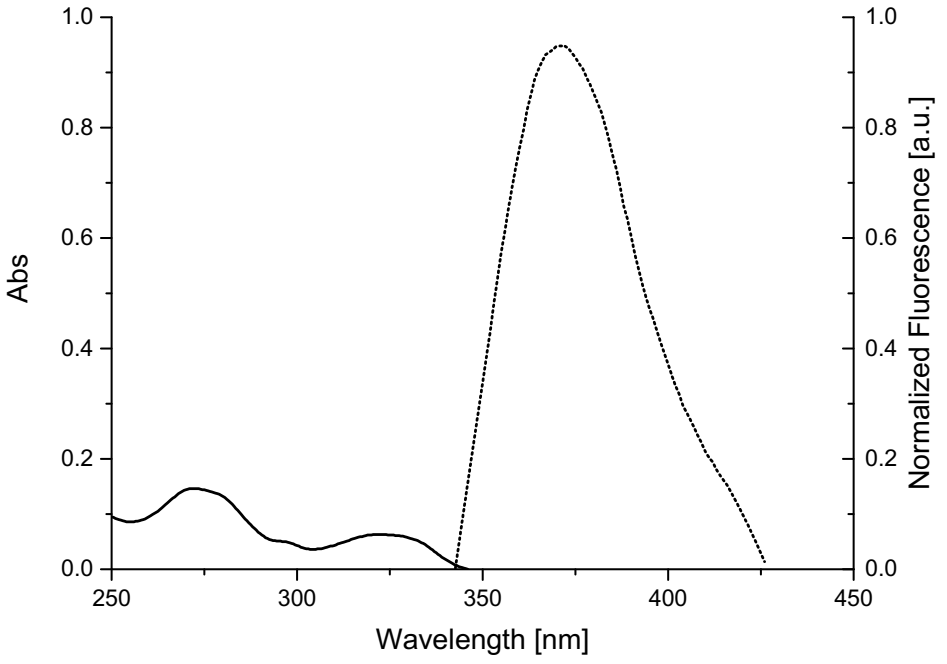


- 1. Absorption (solid trace) and emission (dotted trace) spectra of **2u**


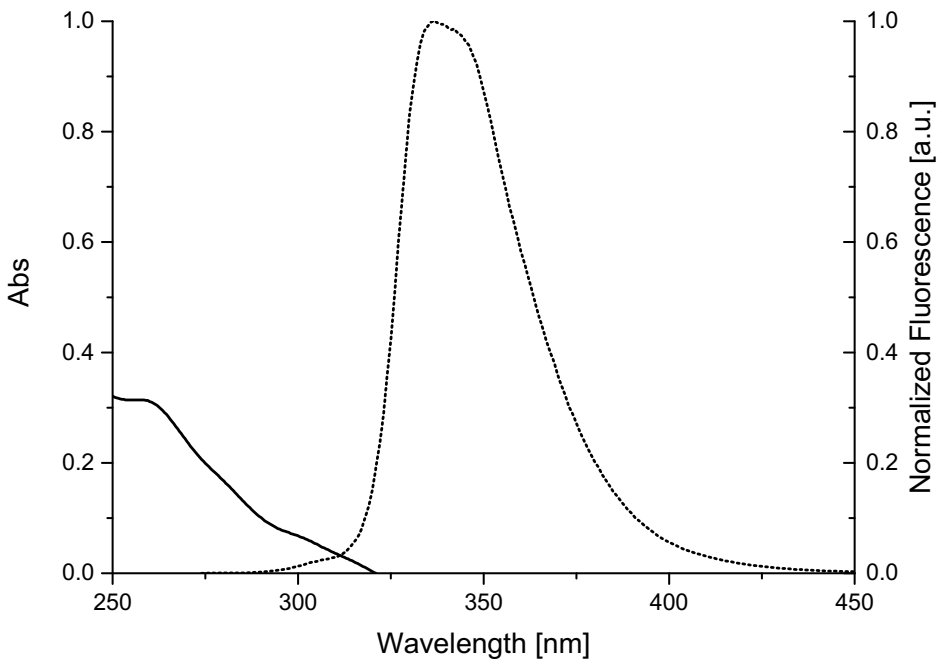


- 1. Absorption (solid trace) and emission (dotted trace) spectra of **2af**


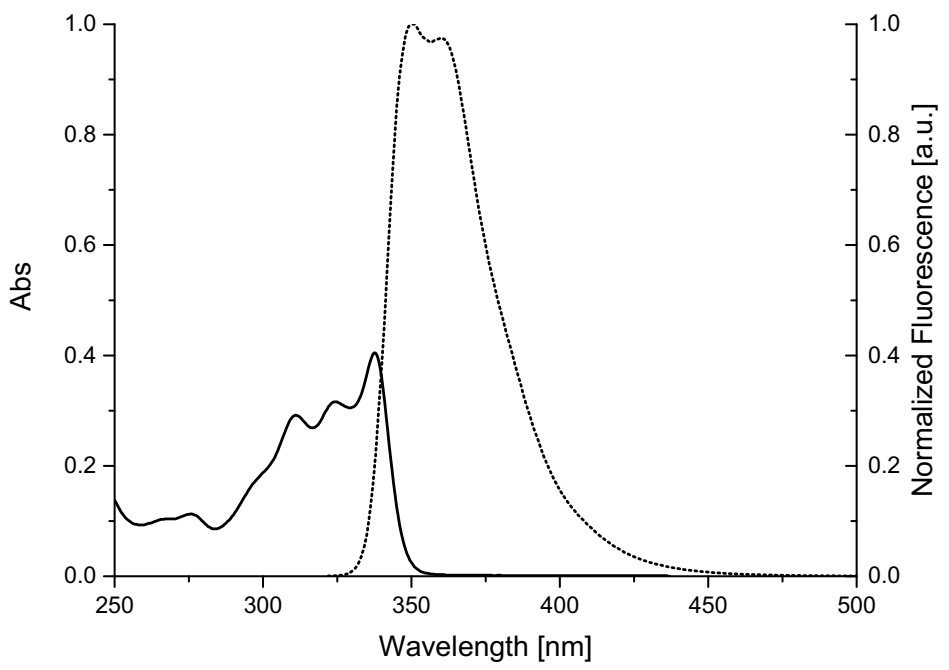


1. **Computational Detailes**

In order to get deeper insight into the two reaction pathways of five-membered palladacycle intermediate, density functional theory (DFT) calculations were carried out using the Gaussian 09 software package.^3,4^ The stationary structures were optimized using PBE0 method and combined basis set. That is, LANL2DZ for Pd atom, and 6-31G(d) basis set for all the other atoms.^5^ Truhlar and coworkers’ SMD solvation model was employed to consider the solvent effect of diethyl ether and hexane.^6^ The geometry optimizations were performed without symmetry constraints, and the nature of the extrema was checked by analytical frequency calculations. The intrinsic reaction coordinate (IRC)^7^ pathways have been traced to verify two desired minima connected by the transition states.

**Table S4. Coordination data sets and free energies for DFT optimized structures at the SMD (Et_2_O/Hexane=1:1) PBE0/combined basis set level at 353 K.**

**A**

-3512.840676

C 3.56857500 4.12888400 0.48964100

C 2.50556800 4.80359000 1.08087800

C 1.26188200 4.19071800 1.13634800

C 1.02491400 2.91425200 0.59465000

C 2.09915600 2.24735400 -0.03309100

C 3.35779800 2.86824900 -0.04781800

P -0.67803100 2.19913500 0.79297900

C 1.98470600 0.87577500 -0.71300800

N 1.77591500 0.99788300 -2.17630300

S 0.43482900 0.34420800 -2.94256600

C 0.78584000 -1.47099700 -3.13980400

C 1.03924100 -2.19321100 -1.82792500

C -0.46604500 -2.00232900 -3.83864200

C 1.99195600 -1.53796800 -4.06991500

O -0.81691800 0.44471100 -2.04972800

C 3.18567200 0.01077400 -0.35364900

C 2.31931400 2.15740700 -2.88478900

C 4.22750100 -0.25138700 -1.22985800

C 5.34468200 -1.02257900 -0.86742700

C 5.31839900 -1.61221700 0.41076800

C 4.33684600 -1.27133500 1.37559500

C 3.28120400 -0.46670200 0.95421800

C 4.46801500 -1.67331900 2.85684400

C 6.50977900 -1.09238300 -1.88067400

O 6.28073600 -2.52873200 0.77748200

C 6.04430300 -3.82919500 0.26180800

C 6.11470000 -1.95413300 -3.09000000

C 7.84244300 -1.60362500 -1.31172600

C 6.80260000 0.33660000 -2.38479200

C 5.79546700 -1.11608200 3.40070000

C 3.34010400 -1.07183600 3.70542500

C 4.42448900 -3.19424300 3.07425500

C -1.72900500 3.34148500 -0.31957500

C -1.13283700 2.76385000 2.52554900

C -2.32274900 4.59258700 0.34393400

C -3.21952400 5.36348900 -0.62808300

C -2.47064100 5.76226700 -1.89450900

C -1.85361000 4.53771000 -2.56057600

C -0.96825400 3.75492200 -1.58885100

C -2.51223900 2.21854100 2.92486200

C -2.94068800 2.75865700 4.28959800

C -1.90311200 2.44227900 5.36314400

C -0.52429300 2.96149300 4.96405500

C -0.09031600 2.42505300 3.59857300

H 0.45046500 4.72020300 1.62625500

H 4.19922200 2.33712500 -0.48431100

H 1.08717300 0.38443500 -0.31019300

H 1.00124400 -3.27440100 -2.01292800

H 0.27606600 -1.95863400 -1.07811900

H 2.02434500 -1.95900000 -1.41689200

H -0.27536600 -3.03877100 -4.14150300

H -0.71381400 -1.43462200 -4.74401500

H -1.33954000 -1.99450100 -3.18366800

H 2.21165500 -2.59066200 -4.28452700

H 2.88069100 -1.09861400 -3.60762800

H 1.80623600 -1.03630600 -5.02768400

H 3.40075000 2.21246200 -2.73202600

H 1.87518400 3.10823100 -2.56667700

H 2.15076000 2.03239600 -3.95779400

H 4.17478700 0.15816200 -2.23298700

H 2.49611400 -0.19867800 1.65279800

H 6.82480100 -4.47588700 0.67265400

H 6.10042000 -3.85380700 -0.83273000

H 5.06102100 -4.20990600 0.56356800

H 6.92337900 -1.96115100 -3.83214300

H 5.21487400 -1.56792300 -3.58371200

H 5.91335500 -2.99359800 -2.80671700

H 8.62746000 -1.42695900 -2.05796100

H 7.84616500 -2.67317600 -1.09536700

H 8.12768900 -1.07291200 -0.39778100

H 7.65556800 0.31655300 -3.07403400

H 7.05764400 1.00615000 -1.55435500

H 5.96530700 0.78119900 -2.93194300

H 5.91720400 -1.39488400 4.45545800

H 5.81304200 -0.02084700 3.34174300

H 6.65469600 -1.50451100 2.84682400

H 3.51113600 -1.32825800 4.75760300

H 2.35566700 -1.46980200 3.43065900

H 3.30539900 0.02228800 3.64039200

H 4.35543700 -3.40813700 4.14860500

H 5.32263800 -3.69133400 2.70379800

H 3.54720600 -3.64429100 2.59422600

H -2.56400600 2.69443300 -0.62557000

H -1.20947800 3.85934400 2.49027600

H -1.51394300 5.25919600 0.67641200

H -2.90870200 4.33341500 1.23135800

H -3.62349400 6.25129700 -0.12343000

H -4.08332500 4.73690600 -0.89581500

H -1.67650500 6.47883100 -1.63519500

H -3.14413000 6.27968200 -2.59037600

H -1.25709600 4.83663700 -3.43323000

H -2.64960700 3.88068000 -2.93502000

H -0.56418900 2.87135800 -2.09079600

H -0.11245000 4.38204200 -1.30397400

H -2.47200200 1.12380400 2.96048300

H -3.26743700 2.47149400 2.16953400

H -3.91450400 2.33229800 4.56311000

H -3.08243700 3.84882800 4.22786300

H -1.85314400 1.35260000 5.50332000

H -2.20764600 2.87050000 6.32731300

H 0.22216700 2.68518600 5.72047900

H -0.54327400 4.06194800 4.93351700

H 0.88838700 2.84071300 3.33317300

H 0.03422600 1.33922400 3.66742100

H 4.55842300 4.57660800 0.45315800

H 2.64454300 5.79213100 1.51161000

Pd -1.33819200 -0.14344900 0.12592300

C -1.69461600 -1.12846500 1.82440800

C -2.45783800 -1.74687800 -0.49863800

C -2.81159800 -1.98835700 1.85890300

C -0.86423200 -1.08756100 2.94887200

C -3.55251200 -2.07326800 0.54217300

H -1.80802600 -2.62125700 -0.62776100

H -2.87338900 -1.48595500 -1.48171500

C -3.13774200 -2.68650300 3.02203600

C -1.17390900 -1.80859400 4.10542400

H 0.04285300 -0.48980400 2.92704000

C -4.17386500 -3.43683500 0.26351800

C -4.67452200 -1.01711100 0.51930400

C -2.32695300 -2.58801500 4.15247100

H -4.00956700 -3.33854300 3.03625400

H -0.51182500 -1.75724700 4.96835000

C -5.47792000 -3.56593900 -0.24088100

C -3.45453800 -4.61902700 0.48519600

C -5.62049400 -1.24608400 -0.63696600

H -4.22687500 -0.01805500 0.45067100

H -2.57884700 -3.14250300 5.05393600

C -6.04039300 -4.82412100 -0.47847800

O -6.28439700 -2.50019200 -0.51511900

C -3.99881000 -5.87408800 0.24293400

H -5.09545300 -1.21626900 -1.60233500

C -5.30574600 -5.97475600 -0.23590500

H -7.05650200 -4.86769900 -0.86225600

H -3.41057300 -6.76844700 0.43215400

H -5.25070400 -1.06222900 1.45352900

H -6.41975500 -0.49877200 -0.65746000

H -2.44259400 -4.54250500 0.87752000

H -5.75025400 -6.94891900 -0.42600900

O -3.56516700 -0.64667000 -3.89995300

C -3.86028500 0.52577600 -3.76631900

O -3.09898600 1.40282500 -3.11132600

C -5.11058200 1.15273900 -4.30598600

H -2.27467500 0.95619200 -2.77225000

H -5.69548100 1.58353700 -3.48579700

H -4.85589100 1.97117900 -4.98819700

H -5.70415900 0.40227100 -4.83022300

**Ts1a**

-3512.801177

C 3.48666100 3.98937500 1.33324400

C 2.41281900 4.46925100 2.07511200

C 1.17794800 3.85005500 1.95154800

C 0.95211000 2.76626100 1.08167400

C 2.03837700 2.30219100 0.30527700

C 3.29064300 2.91575900 0.47915500

P -0.75755600 2.02654800 1.10891800

C 1.98614700 1.13286100 -0.69352100

N 1.99226400 1.57233900 -2.10674800

S 0.63975300 1.35047200 -3.10322800

C 0.86693500 -0.32432400 -3.87392700

C 0.95128200 -1.41355500 -2.81943500

C -0.37566200 -0.49302400 -4.74846300

C 2.11898600 -0.24020800 -4.73672800

O -0.59693200 1.18231500 -2.22645900

C 3.12441200 0.15857800 -0.38621000

C 2.76598600 2.74666000 -2.49878000

C 4.10436300 -0.17287200 -1.30807000

C 5.15518700 -1.06048700 -1.01818500

C 5.12336000 -1.68614400 0.24034400

C 4.22663900 -1.27444700 1.25867500

C 3.23309600 -0.36352300 0.90664000

C 4.40012500 -1.71945200 2.72376700

C 6.26752600 -1.20688500 -2.08041800

O 6.00258400 -2.70574200 0.53760800

C 5.61295700 -3.95934900 0.00243200

C 5.74353700 -1.97500000 -3.30325400

C 7.55938700 -1.87776500 -1.59003700

C 6.68860400 0.20444000 -2.53958400

C 5.81872100 -1.35184100 3.19492600

C 3.41919400 -0.99084300 3.65157800

C 4.17689100 -3.22797900 2.91638600

C -1.83310600 3.45716200 0.46541500

C -1.11730200 1.96077600 2.94688700

C -2.32102500 4.49361900 1.48709200

C -3.28008900 5.49333200 0.83410300

C -2.63478600 6.20241900 -0.35198800

C -2.10372700 5.19301400 -1.36490600

C -1.16243000 4.17894900 -0.71230500

C -2.51126900 1.36533500 3.18676600

C -2.83594600 1.28966400 4.67879400

C -1.78336000 0.48135000 5.43239600

C -0.38367900 1.03804000 5.18622000

C -0.06381000 1.13422800 3.69353600

H 0.36318400 4.21304700 2.56894200

H 4.14523400 2.51680800 -0.06008200

H 1.03103200 0.60540700 -0.53964400

H 0.89289200 -2.39340700 -3.31036900

H 0.11830200 -1.34600900 -2.11032100

H 1.89270200 -1.37311400 -2.26335600

H -0.28755000 -1.43351200 -5.30566900

H -0.46866500 0.31557400 -5.48539000

H -1.29810700 -0.53049500 -4.16046100

H 2.26730100 -1.19800800 -5.24989800

H 3.01254400 -0.03835100 -4.13901200

H 2.03307900 0.53494900 -5.50857500

H 3.82047800 2.60035100 -2.24686800

H 2.41867500 3.67578500 -2.02840600

H 2.71299100 2.86697200 -3.58474700

H 4.05066600 0.27869900 -2.29213900

H 2.52200400 -0.03031800 1.65459100

H 6.35118600 -4.68937000 0.34659900

H 5.60275800 -3.95263600 -1.09386200

H 4.61694200 -4.25856400 0.35167400

H 6.52201300 -2.03817300 -4.07457100

H 4.87224300 -1.48202200 -3.75025200

H 5.44650500 -2.99849400 -3.04750600

H 8.32895900 -1.75175700 -2.36247700

H 7.45706200 -2.95062400 -1.41743400

H 7.93471400 -1.42123400 -0.66861800

H 7.49816900 0.12709600 -3.27576100

H 7.05767600 0.80112900 -1.69659800

H 5.87768500 0.76240700 -3.01797500

H 5.95508500 -1.65133000 4.24233200

H 5.98473600 -0.26917400 3.13460100

H 6.58675900 -1.85088500 2.59836100

H 3.62572500 -1.28213300 4.68831400

H 2.37284300 -1.24595300 3.44658900

H 3.52636100 0.09855000 3.59279600

H 4.13346500 -3.46115600 3.98821300

H 4.98841900 -3.82056400 2.48919700

H 3.23110100 -3.55590600 2.46843300

H -2.71711100 2.93607300 0.06976300

H -1.10472000 2.98295100 3.35042300

H -1.46610700 5.04979200 1.89527000

H -2.82492500 4.01588400 2.33421900

H -3.61531000 6.22355400 1.58283800

H -4.17984700 4.95914200 0.49417800

H -1.80512400 6.82922900 0.00883100

H -3.35402800 6.88210600 -0.82795100

H -1.57612100 5.71100400 -2.17750200

H -2.94211600 4.65761600 -1.83063800

H -0.82517800 3.45221700 -1.45731600

H -0.26714600 4.70256500 -0.34811600

H -2.53621600 0.35493000 2.75611000

H -3.28359600 1.94365400 2.66318400

H -3.82946800 0.84290800 4.81653500

H -2.88984300 2.30629200 5.09839300

H -1.82197000 -0.56346000 5.09108900

H -2.00611800 0.47112800 6.50775600

H 0.36921800 0.41107400 5.68231700

H -0.30315500 2.03776800 5.64058900

H 0.93249200 1.57227600 3.55828900

H -0.03694800 0.12457400 3.26017700

H 4.47337500 4.43557400 1.42915700

H 2.53762600 5.30381100 2.76060400

Pd -1.27899900 -0.06504200 0.15079300

C -1.49358600 -1.91770600 1.08978900

C -2.31328800 -1.85315200 -0.59226600

C -2.71358500 -2.50096200 1.47301800

C -0.32959000 -2.27417200 1.79067200

C -3.55996600 -2.27210100 0.24537600

H -1.83344100 -2.70735000 -1.07420900

H -2.51583000 -1.09632200 -1.38081400

C -2.84805700 -3.27365100 2.61546900

C -0.46224500 -3.03923400 2.95523900

H 0.65579400 -1.95750600 1.45914500

C -4.26259200 -3.50580900 -0.29225100

C -4.57246200 -1.13321100 0.40284300

C -1.70554300 -3.50584800 3.38805000

H -3.79742500 -3.73953500 2.87254400

H 0.42645100 -3.28153700 3.53519600

C -5.56239800 -3.43168000 -0.81715900

C -3.64468900 -4.76274500 -0.27025400

C -5.49662900 -1.07700400 -0.79255800

H -4.03089200 -0.18415100 0.51385400

H -1.77259400 -4.10702600 4.29181000

C -6.21962600 -4.57824100 -1.27418000

O -6.26916600 -2.27012800 -0.89772100

C -4.28396100 -5.90515100 -0.73417800

H -4.93354300 -0.93774400 -1.72548700

C -5.58432900 -5.80970000 -1.23179300

H -7.22789200 -4.47112000 -1.66552200

H -3.77575600 -6.86530800 -0.70005400

H -5.17069600 -1.28886800 1.31064000

H -6.22976500 -0.26964400 -0.70398800

H -2.64082700 -4.84138700 0.14197900

H -6.10178600 -6.69534600 -1.59279600

O -3.58814300 0.16967100 -3.45664000

C -3.85879100 1.33567700 -3.22795200

O -2.98015700 2.21612000 -2.76185000

C -5.21384000 1.94576100 -3.43325100

H -2.09166100 1.77661500 -2.61766200

H -5.59364900 2.33673500 -2.48272300

H -5.13862500 2.79256900 -4.12440200

H -5.90592000 1.20239200 -3.83186300

**C**

-3512.851920

C 4.56394900 3.40583900 -0.34069900

C 3.75153000 4.39360800 0.20345700

C 2.40976800 4.11797000 0.42821100

C 1.82117300 2.88100800 0.10446700

C 2.64553900 1.89080900 -0.48271900

C 4.00824100 2.17698100 -0.66214900

P 0.02894200 2.63931500 0.55674400

C 2.17919000 0.49150100 -0.91861300

N 2.02621700 0.37014400 -2.38296100

S 0.55292000 -0.14573000 -3.06846700

C 0.76576600 -1.96380900 -3.39871600

C 0.86325700 -2.74667000 -2.10220800

C -0.50472200 -2.32866500 -4.16729700

C 1.99284500 -2.12277300 -4.28498400

O -0.53205400 -0.10200200 -1.99894200

C 3.11210100 -0.55607600 -0.32161200

C 2.75453300 1.25595200 -3.28525700

C 4.06600000 -1.23456000 -1.06482600

C 4.94159100 -2.17549400 -0.49770400

C 4.74999900 -2.47429500 0.86416800

C 3.88660600 -1.70845200 1.68714500

C 3.07048500 -0.76992000 1.05799700

C 3.91170400 -1.82080600 3.22364300

C 6.05847100 -2.74016100 -1.40414600

O 5.43333700 -3.51899600 1.45182900

C 4.85999300 -4.78666000 1.17805700

C 5.46686000 -3.70240600 -2.44543000

C 7.20783100 -3.44527500 -0.66641500

C 6.71494600 -1.56469700 -2.15803900

C 5.34589600 -1.56669200 3.72213800

C 3.01713900 -0.75936500 3.87555500

C 3.43046200 -3.18925700 3.73114100

C -0.86653600 3.74054800 -0.69089000

C -0.12048900 3.58585900 2.16352800

C -0.81546700 5.25505600 -0.45906800

C -1.66443700 5.99660100 -1.49529500

C -1.23204300 5.66901700 -2.92159900

C -1.24151100 4.16214800 -3.16122400

C -0.40220700 3.42545400 -2.11767200

C -1.58113900 3.57640500 2.63367900

C -1.74408600 4.32083400 3.95983100

C -0.82719800 3.75340400 5.04027300

C 0.62673900 3.73415600 4.57580600

C 0.78649900 2.99447500 3.24727100

H 1.80571900 4.89326400 0.88758200

H 4.65724000 1.39721800 -1.05146800

H 1.17759800 0.32380900 -0.48288100

H 0.84710900 -3.82045200 -2.32890100

H 0.01155200 -2.52574700 -1.45112100

H 1.78836300 -2.53083300 -1.55957700

H -0.45805700 -3.38834100 -4.44615600

H -0.60447300 -1.74732500 -5.09244800

H -1.40647600 -2.17050800 -3.56934200

H 2.10826600 -3.18029500 -4.55175800

H 2.90652000 -1.80169500 -3.77645100

H 1.89596900 -1.55941600 -5.22178900

H 3.83279200 1.09005000 -3.19717500

H 2.54957400 2.32047200 -3.11469500

H 2.48340900 1.01692200 -4.31804700

H 4.12911000 -1.02335100 -2.12726300

H 2.37287300 -0.17707600 1.64209700

H 5.45301500 -5.52425600 1.72651400

H 4.88961000 -5.02931700 0.10928100

H 3.81640700 -4.83893900 1.51199800

H 6.25000100 -4.05067200 -3.13135800

H 4.68967500 -3.21620900 -3.04630700

H 5.01650400 -4.58641100 -1.97953700

H 8.01830500 -3.62821200 -1.38356800

H 6.92928100 -4.41338800 -0.24667200

H 7.61297500 -2.82962400 0.14321600

H 7.54591700 -1.93892000 -2.76843200

H 7.11994300 -0.82186900 -1.46010500

H 6.02789100 -1.05109300 -2.83788200

H 5.37685300 -1.61903200 4.81838700

H 5.69271500 -0.56887600 3.42668000

H 6.04950100 -2.30469500 3.32821300

H 3.12093200 -0.82380200 4.96512000

H 1.95675200 -0.90701600 3.64078200

H 3.29620900 0.25860100 3.57996100

H 3.31624400 -3.15829800 4.82252600

H 4.13936200 -3.98767700 3.50286200

H 2.45380600 -3.45283600 3.30665600

H -1.91266600 3.41061800 -0.60376200

H 0.17268400 4.63155900 1.99928700

H 0.22362100 5.60470100 -0.53763100

H -1.16622200 5.51750000 0.54561600

H -1.60525000 7.07850600 -1.31538700

H -2.71957300 5.71539800 -1.36124200

H -0.21622800 6.05788600 -3.08937800

H -1.88412700 6.17748700 -3.64414900

H -0.86506300 3.93185900 -4.16714900

H -2.27310900 3.78657800 -3.12044600

H -0.44677000 2.34681600 -2.29299700

H 0.64944400 3.72888100 -2.22419300

H -1.90578700 2.53215600 2.74918200

H -2.23940400 4.02129900 1.87606100

H -2.79200000 4.27171100 4.28431600

H -1.51502200 5.38725700 3.81156100

H -1.14305500 2.72715700 5.28006200

H -0.92440500 4.33348500 5.96753200

H 1.26492100 3.26736700 5.33785700

H 0.98647000 4.76833800 4.46161300

H 1.83597100 3.02427800 2.92862600

H 0.52679700 1.93406200 3.37901200

H 5.62410100 3.58347600 -0.50322800

H 4.15920600 5.36513500 0.47199100

Pd -0.71482500 0.47414500 0.85404200

C -2.60057300 -1.70941300 0.88309600

C -3.37745100 -2.32228200 -0.25774100

C -3.73384800 -1.25988600 1.53578200

C -1.30664300 -1.56366600 1.41972200

C -4.68255800 -1.76515200 0.45793800

H -3.33888500 -3.41665500 -0.31273900

H -3.18811700 -1.90898100 -1.25525700

C -3.69547500 -0.63642200 2.78491300

C -1.25144300 -0.87983800 2.67127600

H -0.44526000 -2.11484500 1.04246200

C -5.69077800 -2.81727200 0.85897300

C -5.39105500 -0.66559900 -0.33018200

C -2.43060200 -0.44641800 3.33036800

H -4.58998800 -0.32782100 3.32089200

H -0.31934100 -0.86593800 3.23015300

C -6.91152100 -2.92710200 0.17431500

C -5.45198100 -3.70792900 1.91092300

C -6.24971300 -1.28477800 -1.41136300

H -4.65550200 0.01847000 -0.76826500

H -2.33058200 0.01986700 4.30823900

C -7.85574700 -3.88933700 0.54392100

O -7.27089700 -2.11170400 -0.85651400

C -6.38039500 -4.67414200 2.28002900

H -5.63980700 -1.88120000 -2.10468700

C -7.59092000 -4.75947200 1.59171000

H -8.78958600 -3.93360700 -0.01061200

H -6.16553200 -5.34978300 3.10387900

H -6.02897300 -0.07691700 0.34290200

H -6.78485300 -0.52820600 -1.99256400

H -4.51488200 -3.62415000 2.45737800

H -8.33103800 -5.50559700 1.87092400

O -3.50017100 -0.75405500 -3.58379800

C -3.63069300 0.44330000 -3.39767100

O -2.79376600 1.17178900 -2.66596500

C -4.74519800 1.26827600 -3.97055600

H -2.01181900 0.62039600 -2.36978800

H -5.23073400 1.85709200 -3.18550700

H -4.33530800 1.97852800 -4.69817400

H -5.47346100 0.62493100 -4.46666100

**B**

-3512.835349

C -3.02063300 3.03560200 3.14755400

C -1.94596700 3.91058800 3.04839900

C -0.80251300 3.49772100 2.38139000

C -0.66096700 2.21396400 1.81544100

C -1.75285300 1.32028600 1.93087200

C -2.91396200 1.77655600 2.57996900

P 0.96034700 1.92354900 0.93071600

C -1.83635700 -0.11442600 1.37260200

N -1.73457500 -1.15007900 2.41727300

S -0.33750800 -2.14283300 2.50768500

C -0.85871700 -3.74246700 1.70695600

C -1.24106600 -3.51048000 0.25718800

C 0.39497200 -4.61229000 1.80471300

C -1.98822500 -4.33371300 2.53817700

O 0.69401500 -1.59042300 1.55844200

C -3.13383300 -0.25636000 0.56327300

C -2.32804800 -0.93618200 3.72921600

C -4.05901800 -1.25636700 0.81716900

C -5.26764200 -1.38132800 0.11072800

C -5.48261300 -0.46846100 -0.93461700

C -4.63481500 0.64933700 -1.13994500

C -3.45779800 0.70794300 -0.39683900

C -5.06140900 1.83474800 -2.02865700

C -6.25449900 -2.46751600 0.59562700

O -6.56162400 -0.61851400 -1.78199500

C -6.32674100 -1.57263400 -2.80465700

C -5.70194200 -3.86336900 0.26646700

C -7.68523500 -2.35867600 0.04646000

C -6.39603800 -2.34825100 2.12751800

C -4.03301400 2.97190500 -1.97537600

C -5.25766400 1.46221600 -3.50667000

C -6.38600300 2.39534200 -1.47934200

C 2.23403700 1.97979700 2.31314200

C 1.21884700 3.55491400 0.03989100

C 2.44693400 3.29965100 3.06288500

C 3.63047100 3.18225700 4.02789500

C 3.43182100 2.04088000 5.02094900

C 3.17066500 0.72384700 4.29663900

C 2.00794100 0.83663800 3.30991700

C 2.54874300 3.52214900 -0.72602300

C 2.78174200 4.81032400 -1.51642700

C 1.61639200 5.10454400 -2.45625400

C 0.30285600 5.17037300 -1.68324100

C 0.05051100 3.88510400 -0.89507600

H 0.00236700 4.21611600 2.28153000

H -3.77725500 1.11894300 2.61457900

H -0.98185200 -0.27867700 0.70126000

H -1.43042800 -4.47654400 -0.22858200

H -0.42794800 -3.01731200 -0.28550900

H -2.14628900 -2.90331400 0.16252900

H 0.17160100 -5.61361700 1.41688500

H 0.73713400 -4.72843700 2.84131400

H 1.21429400 -4.19321100 1.21219000

H -2.27005100 -5.31085100 2.12652700

H -2.88121200 -3.70071700 2.52964500

H -1.68732500 -4.49407800 3.58146100

H -3.41496200 -0.82912900 3.64589800

H -1.92932200 -0.05921300 4.25700500

H -2.14461700 -1.81671500 4.35253600

H -3.82901400 -1.96590900 1.60270600

H -2.77418700 1.53545700 -0.54292000

H -7.22846600 -1.60324500 -3.42306800

H -6.14095800 -2.57280600 -2.39562900

H -5.46867000 -1.29337900 -3.42860100

H -6.37352600 -4.64129200 0.65249900

H -4.71379400 -4.02544200 0.71312900

H -5.60142800 -4.01645000 -0.81431100

H -8.32081700 -3.06156400 0.60027100

H -7.76988400 -2.62000100 -1.01033900

H -8.10317400 -1.35585900 0.18135500

H -7.11212200 -3.09504900 2.49198800

H -6.77060600 -1.35789100 2.41356700

H -5.45695600 -2.51850700 2.66313500

H -4.39285000 3.80971700 -2.58445600

H -3.05540100 2.67244300 -2.37173700

H -3.88886400 3.34877500 -0.95613600

H -5.39725700 2.37504500 -4.10010900

H -6.14024400 0.83855500 -3.65860200

H -4.38585300 0.93758600 -3.91413000

H -6.70953100 3.25615800 -2.07930000

H -6.26840000 2.73531900 -0.44308900

H -7.18154900 1.64523500 -1.50757900

H 3.16756200 1.76460200 1.77448100

H 1.29061200 4.35902000 0.78417400

H 1.54867300 3.55007000 3.64216700

H 2.62864000 4.12919400 2.36840700

H 3.76691100 4.13362500 4.55933200

H 4.55232400 3.01031800 3.45223700

H 2.57496700 2.27445700 5.67103800

H 4.30706700 1.95129400 5.67789400

H 2.96338600 -0.07556400 5.02014600

H 4.07626200 0.41999500 3.75078700

H 1.86780100 -0.10941200 2.77722500

H 1.07816700 1.03067800 3.86666900

H 2.54164700 2.66941800 -1.41886000

H 3.38483100 3.35208600 -0.03542400

H 3.71967000 4.72717700 -2.08111300

H 2.91089300 5.65275700 -0.81992600

H 1.55167300 4.30758400 -3.21293800

H 1.78949600 6.04267400 -2.99952100

H -0.53807600 5.35430400 -2.36502100

H 0.33164200 6.02359600 -0.98878900

H -0.87793000 3.97912900 -0.31950800

H -0.09896700 3.05427600 -1.59544000

H -3.94074500 3.33385700 3.64370800

H -1.99918100 4.91299200 3.46576500

Pd 1.37864600 0.08784100 -0.69899200

C 1.89343700 -1.42505100 -1.94944100

C 3.21959300 -0.41978300 0.01326400

C 3.23623000 -1.85018400 -1.94226200

C 0.97725700 -2.14817000 -2.72254700

C 4.13939300 -0.98634000 -1.08714300

H 2.97136400 -1.18302600 0.75660400

H 3.71028500 0.41893800 0.51919900

C 3.64075900 -2.96365600 -2.67831500

C 1.38427100 -3.25545500 -3.47374500

H -0.07447200 -1.86595000 -2.73486900

C 5.32331300 -1.75019300 -0.49780200

C 4.69449300 0.18170600 -1.93032400

C 2.71418200 -3.66433900 -3.45203000

H 4.67461000 -3.30198700 -2.63429400

H 0.65514500 -3.80582800 -4.06653500

C 6.64067600 -1.52347100 -0.92923200

C 5.13738600 -2.72060900 0.49621900

C 5.88361600 -0.24105800 -2.75649200

H 3.90858600 0.57280000 -2.58632200

H 5.00868900 0.99748200 -1.26469600

H 3.03074500 -4.53289900 -4.02577300

C 7.72063300 -2.19147700 -0.34256600

O 6.96523400 -0.64836900 -1.92487500

C 6.20037100 -3.40226500 1.07512700

H 4.12454700 -2.94528000 0.82098000

H 6.27748200 0.58475600 -3.35587900

H 5.61968000 -1.06411400 -3.43682400

C 7.50323900 -3.12253300 0.66115200

H 8.72082300 -1.96600700 -0.70366500

H 6.01469000 -4.14507900 1.84674500

H 8.34803300 -3.63993600 1.10975800

O -0.51098000 0.53570500 -2.02268100

C -0.20064600 0.91435600 -3.15119600

O 1.04586500 1.20946500 -3.48189300

C -1.17136200 1.08700600 -4.26998700

H 1.61034900 0.92995300 -2.70049100

H -1.21262500 2.14066600 -4.56778600

H -0.82869000 0.51526200 -5.13884600

H -2.15923400 0.74512400 -3.96320600

**Ts1b**

-3512.810754

C -2.31517900 -4.53512200 0.99017200

C -1.13011000 -4.85638700 1.63842900

C -0.02936300 -4.02785000 1.47703800

C -0.05225600 -2.86145500 0.68492000

C -1.28221200 -2.49955900 0.07642200

C -2.37479800 -3.36845100 0.24479600

P 1.62487400 -2.07982900 0.42948500

C -1.60918700 -1.19023700 -0.67968300

N -1.68557100 -1.35308400 -2.13910600

S -0.56646800 -0.57559500 -3.17999900

C -1.46369300 0.89647600 -3.89402400

C -1.78723600 1.90886800 -2.81018500

C -0.43971800 1.47146900 -4.87432700

C -2.68673500 0.38854900 -4.64394400

O 0.49498100 0.06008200 -2.32056700

C -2.91923100 -0.61527300 -0.10387800

C -2.25914300 -2.55594200 -2.71639300

C -3.95922100 -0.19592400 -0.91958600

C -5.16125300 0.33796900 -0.42355100

C -5.25789500 0.48247700 0.97055100

C -4.30515500 -0.09534000 1.84531300

C -3.13649900 -0.59785300 1.27870200

C -4.59512600 -0.28100300 3.34795500

C -6.27185900 0.66050900 -1.44764400

O -6.31709600 1.15928500 1.53856700

C -6.15050400 2.56797000 1.52480100

C -5.89154400 1.90338900 -2.26682100

C -7.67187100 0.86859300 -0.84971900

C -6.41256500 -0.53317600 -2.41471200

C -5.95240900 -0.98883500 3.51639800

C -3.54974000 -1.18620900 4.01324300

C -4.61177000 1.04928800 4.11674300

C 2.36192200 -3.22617600 -0.86863300

C 2.62741100 -2.50909300 1.95428100

C 2.47934600 -4.70556500 -0.48705500

C 3.18583500 -5.49843500 -1.59067300

C 2.47799300 -5.34985700 -2.93360500

C 2.33834000 -3.87900200 -3.31100000

C 1.64702400 -3.06992000 -2.21359200

C 4.08098500 -2.04443900 1.76572900

C 4.94097100 -2.44445100 2.96634600

C 4.36602400 -1.91398700 4.27649000

C 2.91466600 -2.34976300 4.45482900

C 2.05842100 -1.93803800 3.25686200

H 0.88416100 -4.31284500 1.98504100

H -3.32600200 -3.08832400 -0.19812900

H -0.79841800 -0.46954800 -0.50473500

H -2.19506800 2.81846600 -3.26955200

H -0.88220300 2.18788300 -2.26115500

H -2.51998600 1.53363300 -2.09098700

H -0.88378200 2.32485000 -5.40090800

H -0.13644900 0.73696600 -5.63120700

H 0.45700500 1.82146600 -4.35293800

H -3.19071500 1.22940700 -5.13656400

H -3.41246200 -0.09235200 -3.98095800

H -2.41160600 -0.32835400 -5.42803600

H -3.33384800 -2.61735200 -2.50538400

H -1.78227800 -3.47806700 -2.35783400

H -2.14699600 -2.52484500 -3.80465900

H -3.82881700 -0.29421500 -1.98975700

H -2.38143500 -1.02627900 1.92555900

H -6.99700400 2.98853800 2.07528100

H -6.15322900 2.97085300 0.50488200

H -5.21429300 2.86901000 2.01069400

H -6.66462500 2.11801300 -3.01630000

H -4.94325300 1.76276000 -2.79788400

H -5.78516700 2.79201600 -1.63425200

H -8.39901900 0.89692900 -1.67144100

H -7.77595100 1.80476400 -0.29837000

H -7.95760900 0.04941100 -0.18199000

H -7.22645700 -0.33771100 -3.12353200

H -6.65529700 -1.45562800 -1.87336800

H -5.51155900 -0.71689100 -3.00820000

H -6.15804800 -1.15430200 4.58193400

H -5.94473200 -1.96862400 3.02311500

H -6.77331600 -0.40108400 3.09873200

H -3.84298500 -1.36710200 5.05429200

H -2.55053400 -0.73821100 4.03579200

H -3.47358400 -2.16203000 3.51875000

H -4.66441700 0.85377600 5.19581600

H -5.47649600 1.66425000 3.85729500

H -3.70146800 1.63149700 3.92969000

H 3.37206300 -2.81870600 -0.99792100

H 2.65366600 -3.60288300 2.04451500

H 1.47971800 -5.13183500 -0.32902700

H 3.03439100 -4.83285300 0.45010100

H 3.24212600 -6.55614800 -1.30059600

H 4.22353600 -5.14441500 -1.68395200

H 1.47857100 -5.80634900 -2.86795900

H 3.02145700 -5.89808000 -3.71452800

H 1.78010300 -3.77335700 -4.25079500

H 3.33502000 -3.45026700 -3.49190100

H 1.62639900 -2.01349900 -2.49267300

H 0.60481200 -3.40913100 -2.11478000

H 4.09993300 -0.95175900 1.64583100

H 4.51277000 -2.46733500 0.84990800

H 5.96428100 -2.07500300 2.82021100

H 5.00908800 -3.54201300 3.01572000

H 4.41352300 -0.81510400 4.27413800

H 4.97551400 -2.25262300 5.12448400

H 2.49515200 -1.91479200 5.37123600

H 2.87191300 -3.44266600 4.58039200

H 1.02291700 -2.26796100 3.40809900

H 2.03477300 -0.84366600 3.19033200

H -3.19410200 -5.16786900 1.08317200

H -1.05344500 -5.74384600 2.26142700

Pd 2.14590300 0.16384700 -0.14200400

C 1.11793600 1.15572600 1.26722200

C 2.64838400 2.25078200 -0.66195100

C 1.41686800 2.52794200 1.43114500

C 0.14960300 0.59889000 2.11145000

C 2.47964300 3.16434200 0.55618200

H 1.70095000 2.18237400 -1.21600900

H 3.33410100 2.67627800 -1.41002900

C 0.77858800 3.27285200 2.42826500

C -0.47738100 1.34831300 3.10690700

H -0.12092300 -0.44763900 2.00735600

C 2.12298000 4.56997900 0.08414400

C 3.78392200 3.26390100 1.37452500

C -0.16011400 2.69172000 3.27228000

H 1.01902500 4.32916600 2.53601600

H -1.20822700 0.87781300 3.75929400

C 3.09016300 5.58715500 0.03996900

C 0.83602100 4.88390300 -0.37006300

C 4.82574400 4.08485900 0.65067600

H 4.17522400 2.26121900 1.58207300

H -0.64337900 3.28413200 4.04600500

C 2.76396700 6.86932700 -0.41428500

O 4.38255900 5.42373200 0.44403200

C 0.50477600 6.15113600 -0.83339700

H 5.08944500 3.63970000 -0.31851900

C 1.47772300 7.15152200 -0.84850800

H 3.54481500 7.62562300 -0.41927700

H -0.50635800 6.35987600 -1.17345500

H 3.57365000 3.74059800 2.34088900

H 5.74412400 4.17457000 1.23857000

H 0.07369000 4.10812400 -0.33941900

H 1.23528200 8.15113200 -1.20151200

O 3.64352800 -0.64144700 -1.59628900

C 4.76128900 -0.09955100 -1.42820800

O 4.93456500 0.91006000 -0.65792700

C 5.97385200 -0.62753500 -2.13890900

H 3.76738500 1.35372400 -0.46480700

H 6.68851400 -1.00867800 -1.40013600

H 5.70708500 -1.42639000 -2.83325600

H 6.46910600 0.18682500 -2.67749600

**D**

-3512.843727

C 3.74118300 3.89422700 -1.44554000

C 2.67988200 4.79190200 -1.39930300

C 1.40358200 4.31677400 -1.13225200

C 1.13731700 2.95132100 -0.92537100

C 2.20493700 2.03771400 -1.04309900

C 3.49453400 2.54070200 -1.27112800

P -0.58999100 2.51418900 -0.40418900

C 2.03522200 0.51377900 -1.02988500

N 1.78934700 0.01610500 -2.40461900

S 0.33089300 -0.73476500 -2.82851100

C 0.67546300 -2.55581500 -2.66579200

C 1.25234000 -2.95893100 -1.32467300

C -0.67958600 -3.21507000 -2.91113200

C 1.64589400 -2.85301700 -3.80634200

O -0.79965500 -0.38967500 -1.85699400

C 3.21400000 -0.16712600 -0.34785700

C 2.35437800 0.75372300 -3.53607500

C 4.17619600 -0.88644500 -1.03904700

C 5.26224400 -1.50487600 -0.39789300

C 5.29197500 -1.42637300 1.00685900

C 4.41426600 -0.58276200 1.73365400

C 3.37151700 0.01447900 1.02761300

C 4.64067200 -0.25173200 3.22160200

C 6.32996700 -2.16333200 -1.29929200

O 6.20636200 -2.16866100 1.72536400

C 5.81243900 -3.52087700 1.89585800

C 5.76768300 -3.44018300 -1.94336300

C 7.66011500 -2.49801900 -0.60662600

C 6.69480000 -1.17372500 -2.42498000

C 3.67178800 0.83493200 3.70634500

C 4.44985500 -1.46809600 4.14130900

C 6.06602500 0.30095900 3.39809200

C -1.63976600 3.16420500 -1.82742600

C -0.85472900 3.81773000 0.92292400

C -1.00738700 2.94303900 -3.20704600

C -1.89177500 3.50373100 -4.32222700

C -3.29674800 2.90999500 -4.28712300

C -3.93067500 3.09503100 -2.91233800

C -3.03916100 2.53294400 -1.80479700

C -2.27332200 3.78064500 1.50820100

C -2.45607400 4.84856300 2.58847800

C -1.40689000 4.72685500 3.68856900

C -0.00031600 4.78523900 3.10135500

C 0.21028000 3.72152800 2.02389200

H 0.59219800 5.03739200 -1.07652700

H 4.32627300 1.84335900 -1.31445900

H 1.14083700 0.27886600 -0.43545900

H 1.37685700 -4.04985000 -1.31252500

H 0.58194600 -2.70089900 -0.50264400

H 2.23184000 -2.50907700 -1.14572000

H -0.53310700 -4.29830400 -2.99821500

H -1.15038500 -2.86916900 -3.84025800

H -1.36403300 -3.02994000 -2.07906500

H 1.86018100 -3.92836200 -3.81315500

H 2.59852100 -2.32813700 -3.67649100

H 1.23011200 -2.59277300 -4.78728700

H 3.43777800 0.83550300 -3.41291600

H 1.94071000 1.76271600 -3.65712900

H 2.17885100 0.19173500 -4.45760300

H 4.06904500 -0.98690100 -2.11430400

H 2.64201100 0.62291100 1.55167700

H 6.56533700 -3.98905400 2.53644600

H 5.77040300 -4.06043100 0.94222100

H 4.82931900 -3.59740500 2.37591700

H 6.51273100 -3.89023000 -2.61232400

H 4.86981100 -3.23374500 -2.53798700

H 5.49908300 -4.19106500 -1.19176800

H 8.38960400 -2.77681300 -1.37770500

H 7.59503400 -3.33672600 0.08876700

H 8.06390900 -1.63897600 -0.06150500

H 7.47344800 -1.61019900 -3.06256300

H 7.08593000 -0.23487300 -2.01466700

H 5.84977200 -0.92962000 -3.07642300

H 3.92017900 1.09127000 4.74350100

H 2.62452900 0.51313900 3.68863900

H 3.75689000 1.75562500 3.11599800

H 4.46161500 -1.13908400 5.18859500

H 5.24724100 -2.20578100 4.02668600

H 3.48463700 -1.95748100 3.96437300

H 6.23608800 0.56669000 4.44967700

H 6.21088900 1.20805400 2.79829500

H 6.82508500 -0.42968000 3.10614400

H -1.73692000 4.24981300 -1.66455600

H -0.73740800 4.79107500 0.42270500

H -0.86887400 1.86438400 -3.36360700

H -0.01692900 3.40822900 -3.25800800

H -1.41863000 3.31453200 -5.29489000

H -1.95536300 4.59741100 -4.21575300

H -3.24191100 1.83550900 -4.51728700

H -3.92394900 3.36605700 -5.06414300

H -4.91221700 2.60447100 -2.87499400

H -4.10921500 4.16569600 -2.73048900

H -3.52514600 2.67209300 -0.83328500

H -2.92770700 1.44996000 -1.94319800

H -2.46696100 2.78825900 1.93245000

H -3.01625100 3.94568100 0.71904400

H -3.46675900 4.76932900 3.01013500

H -2.38638200 5.84770200 2.13126000

H -1.54116900 3.77098600 4.21605300

H -1.54276000 5.51922600 4.43640800

H 0.75255200 4.65803500 3.89033200

H 0.16872300 5.78285900 2.66720700

H 1.20999500 3.83683600 1.58878400

H 0.18922700 2.72374800 2.47719000

H 4.75541100 4.24277900 -1.62314900

H 2.84365800 5.85564900 -1.55207600

Pd -1.22876400 0.15798400 0.20231800

C -1.81945400 -1.70685900 0.72615000

C -3.96081300 -1.37402300 -1.62262400

C -3.08889700 -2.30568300 0.54685800

C -0.82338200 -2.40796300 1.41545300

C -4.27810100 -1.59042200 -0.13745700

H -3.76633100 -2.31675100 -2.14447600

H -4.79392000 -0.87038200 -2.12923500

C -3.26408500 -3.61091700 1.03474100

C -1.02403100 -3.70349200 1.89124700

H 0.13115600 -1.92454000 1.60956800

C -5.58949900 -2.36158200 0.02065900

C -4.52790600 -0.22372600 0.52382000

C -2.25463600 -4.31147700 1.68828300

H -4.22982000 -4.09256600 0.91416300

H -0.22482000 -4.21878200 2.42055800

C -6.47324000 -2.05391400 1.07154100

C -5.98269700 -3.37193200 -0.86574100

C -5.00129700 -0.39539200 1.94551700

H -3.61309900 0.37635600 0.52060500

H -5.28913800 0.32172800 -0.05140200

H -2.44571900 -5.31918000 2.05069300

C -7.68920400 -2.73243200 1.21109500

O -6.24823800 -1.08932100 2.00208500

C -7.18433400 -4.05849600 -0.72997300

H -5.32148100 -3.63616300 -1.68625600

H -5.18398500 0.56730900 2.43187200

H -4.25970500 -0.94371700 2.54052600

C -8.04302100 -3.73226200 0.31868700

H -8.33884700 -2.45240200 2.03617200

H -7.44807400 -4.83943800 -1.43847800

H -8.98869000 -4.25506700 0.44116500

O 0.31258800 0.30980500 2.80825200

C -0.89440900 0.45288300 3.03663500

O -1.81433500 0.56955500 2.13757600

C -1.41520300 0.47039600 4.45737400

H -3.06184700 -0.76134600 -1.73002600

H -0.65709800 0.86839300 5.13727700

H -2.34422700 1.03997100 4.54567200

H -1.62849400 -0.56473400 4.75324100

1. **^1^H , ^19^F, ^13^C NMR and HPLC Spectra**

1. **References**
2. Huang, Q.; Fazio, A.; Dai, G.; Campo, M. A.; Larock, R. C. Pd-Catalyzed Alkyl to Aryl Migration and Cyclization:  An Efficient Synthesis of Fused Polycycles via Multiple C−H Activation. *J. Am. Chem. Soc.* **2004**, *126*, 7460−7461.
3. Ye, J.; Shi, Z.; Sperger, T.; Yasukawa, Y.; Kingston, C.; Schoenebeck, F.; Lautens, M. Remote C−H alkylation and C−C bond cleavage enabled by an in situ generated palladacycle. *Nat. Chem.* **2017**, *9*, 361−368.
4. (a) Becke, A. D. Density-functional thermochemistry. III. The role of exact exchange. *J. Chem. Phys.* **1993**, *98*, 5648−5652. (b) Stephens, P. J.; Devlin, F. J.; Chabalowski, C. F.; Frisch, M. J. Ab Initio Calculation of Vibrational Absorption and Circular Dichroism Spectra Using Density Functional Force Fields. *J. Phys. Chem.* **1994**, *98*, 11623−11627. (c) Lee, C.; Yang, W.; Parr, R. G. Development of the Colle-Salvetti correlation-energy formula into a functional of the electron density. *Phys. Rev. B* **1988**, *37*, 785−789.
5. Frisch, M. J.; Trucks, G. W.; Schlegel, H. B.; Scuseria, G. E.; Robb, M. A.; Cheeseman, J. R.; Scalmani, G.; Barone, V.; Mennucci, B.; Petersson, G. A.; Nakatsuji, H.; Caricato, M.; Li, X.; Hratchian, H. P.; Izmaylov, A. F.; Bloino, J.; Zheng, G.; Sonnenberg, J. L.; Hada, M.; Ehara, M.; Toyota, K.; Fukuda, R.; Hasegawa, J.; Ishida, M.; Nakajima, T.; Honda, Y.; Kitao, O.; Nakai, H.; Vreven, T.; Montgomery, J. A., Jr.; Peralta, J. E.; Ogliaro, F.; Bearpark, M.; Heyd, J. J.; Brothers, E.; Kudin, K. N.; Staroverov, V. N.; Kobayashi, R.; Normand, J.; Raghavachari, K.; Rendell, A.; Burant, J. C.; Iyengar, S. S.; Tomasi, J.; Cossi, M.; Rega, N.; Millam, J. M.; Klene, M.; Knox, J. E.; Cross, J. B.; Bakken, V.; Adamo, C.; Jaramillo, J.; Gomperts, R.; Stratmann, R. E.; Yazyev, O.; Austin, A. J.; Cammi, R.; Pomelli, C.; Ochterski, J. W.; Martin, R. L.; Morokuma, K.; Zakrzewski, V. G.; Voth, G. A.; Salvador, P.; Dannenberg, J. J.; Dapprich, S.; Daniels, A. D.; Farkas, Ö.; Foresman, J. B.; Ortiz, J. V.; Cioslowski, J.; Fox, D. J. *Gaussian 09*, Revision A.02; Gaussian, Inc.: Wallingford, CT, 2009.
6. Hay, P. J.; Wadt, W. R. Ab initio effective core potentials for molecular calculations. Potentials for the transition metal atoms Sc to Hg. *J. Chem. Phys.* **1985**, *82,* 270−283.
7. Marenich, A. V.; Cramer,C. J.; Truhlar, D. G. Universal Solvation Model Based on Solute Electron Density and on a Continuum Model of the Solvent Defined by the Bulk Dielectric Constant and Atomic Surface Tensions. *J. Phys. Chem. B* **2009**, *113*, 6378−6396.
8. Fukui, K. Formulation of the reaction coordinate. *J. Phys. Chem.* **1970**, *74*, 4161−4163.
